# The inclusion of pregnant women in research during disease outbreaks globally: A scoping review

**DOI:** 10.1590/1518-8345.7517.4599

**Published:** 2025-07-11

**Authors:** Haley Kartchner Williams, Maria Docal, Angela Chang Chiu, Catherine Ling, Nancy R Reynolds

**Affiliations:** 1 Johns Hopkins University, School of Nursing, Baltimore, Maryland, United States of America

**Keywords:** Pregnant Women, Pregnancy, Disease Outbreaks, Biomedical Research, Clinical Studies as Topic, Eligibility Determination

## Abstract

this scoping review aims to search the literature about conducting research on pregnant women during outbreaks globally and synthesize the findings to identify themes, analyze knowledge gaps, and provide evidence-based recommendations to inform future research.

a comprehensive literature search was conducted in November 2023 using PubMed, Embase, CINAHL, Web of Science, Scopus, and Global Health databases. Key terms included “pregnancy,” “disease outbreaks,” “biomedical research,” and “eligibility criteria.” Twenty-three articles published between 2017 and 2023 were included in the synthesis.

this review identified a pattern of exclusion of pregnant women from research during multiple disease outbreaks across diverse regions. The exclusion rates ranged from 52% to 97.8%. Ethical and moral concerns were raised, emphasizing the need for a shift toward the presumptive inclusion of pregnant women in research. Recommendations emerged from discussions on the risks and benefits of research participation, global institutional and strategic changes, and the standardized collection of pregnancy-specific data to inform public health responses.

this scoping review highlights the systemic exclusion of pregnant women from research during disease outbreaks, underscoring ethical concerns, critical knowledge gaps, and structural barriers while providing a foundation for advancing maternal health research during public health emergencies.

## Highlights

**(1)** Pregnant women have been excluded from research during outbreaks globally. **(2)** Automatic ineligibility of pregnant women violates ethical principles. **(3)** Literature recommends a shift to the presumptive inclusion of pregnant women in research. **(4)** Surveillance systems collecting pregnancy-specific data should be strengthened.**(5)** Global institutional and strategic changes are needed to prioritize pregnant women.

## Introduction

Pregnant women represent an important research subpopulation, with 140 million women worldwide giving birth each year^(1)^. However, pregnant women have a long history of systematic exclusion from biomedical research globally. This pattern of “protection by exclusion” has limited the participation of pregnant women and even women of childbearing age in clinical therapeutic trials, drug development, and vaccine development trials^(2)^. The most frequently cited reason for exclusion is the protection of the fetus^(2)^. While the potential risks of conducting research involving pregnant women must be carefully considered, automatic ineligibility denies the mother and baby any potential benefit of an intervention and raises substantial ethical concerns^(2)^.

In the 1990s, prominent reports highlighted the underrepresentation of pregnant women in the research agenda, leading to significant policy and legislative changes^(3)^. However, despite increased efforts to ensure inclusion, these recommendations have not been widely implemented, and pregnant women remain significantly underrepresented as trial participants, particularly for interventional and drug trials^(4-5)^. Consequently, most current treatments for medical conditions have never been studied in pregnancy, leading physicians to prescribe treatments and medications “off-label” without rigorous clinical trials or sufficient supporting evidence of safety and efficacy in pregnant women^(2,6)^.

Disease outbreaks and pandemics present critical opportunities for conducting clinical research, as they are often the only periods with sufficient infected patients to conduct clinical trials for vaccines and treatments^(7)^. Notably, pregnant women face a higher risk of severe disease and increased mortality and morbidity than the general population for multiple outbreak-prone diseases, including Ebola virus (EVD), Zika virus (ZIKV), hepatitis E, Lassa fever, and influenza^(7-8)^. EVD is perhaps the most striking example, with Ebola-related maternal mortality estimated at approximately 90% and fetal and newborn loss approaching 100%^(6)^. ZIKV can be transmitted from mother to fetus, leading to growth restriction, fetal brain anomalies, and fetal loss^(9)^.

A global perspective on this issue is essential, as the risk of future pandemics is higher than ever, posing an impending threat to global health security^(10)^. Seventy percent of health workers and first responders worldwide are capable of becoming pregnant, and their inclusion in research during outbreaks is crucial for informing public health responses^(11)^. Public health challenges are further amplified in low- and middle-income countries (LMICs), which may lack resources to respond to outbreaks and reach women living in humanitarian settings, such as refugee camps and conflict zones.

This scoping review was conducted following the Joanna Briggs Institute (JBI) framework for scoping reviews^(12)^ and aims to examine the literature on research involving pregnant women during outbreaks globally. It synthesizes the findings to identify themes, analyze knowledge gaps, and provide evidence-based recommendations for future research. The intended audience includes healthcare professionals, researchers, policymakers, and global health organizations responsible for maternal and child health. This review is needed to address ethical concerns and the lack of pregnancy-specific data, which forces clinicians to rely on unverified assumptions, thereby jeopardizing maternal and fetal health outcomes^(2,6)^. Conducting this review is justified as it provides actionable recommendations to promote equity and improve health outcomes during public health emergencies.

## Methods

### Study design

This study is a scoping review and received no external funding. To ensure fidelity and rigor, the key components of the JBI framework for scoping reviews were used as the methodological foundation for the review^(12)^, with reporting guided by the Preferred Reporting Items for Systematic Reviews and Meta-Analyses Extension for Scoping Reviews (PRISMA-ScR) checklist^(13)^. The search strategy was informed by the research question “how have pregnant women been included in research during disease outbreaks and pandemics globally?”. A comprehensive, non-blinded literature search was conducted by a team of three reviewers in November 2023 in Baltimore, Maryland, USA, using PubMed, Embase, CINAHL, Web of Science, Scopus, and Global Health electronic databases. The search utilized key terms such as: ‘*pregnancy*’ OR ‘*maternal*’ AND ‘*disease outbreaks*’ OR ‘*epidemics*’ OR ‘*pandemics*’ AND ‘*clinical studies*’ OR ‘*research*’ OR ‘*surveillance*’ AND ‘*patient selection*’ OR ‘*research subjects*’ OR ‘*eligibility criteria*.’ A detailed search strategy, including MeSH keywords and associated terms, is outlined in Figure 1. The team used Covidence as a citation management tool to streamline the review process by removing duplicate records, assigning studies to individual reviewers, and organizing study selection and evaluation criteria.

**Figure 1 -:** Search terms

**Date of search**	**Search engine**	**Search terms**	**Retrieved citations**
11/7/2023	PubMed	(((((“Pregnancy”[Mesh]) OR “Obstetrics”[Mesh]) OR “Pregnant Women”[Mesh] OR pregnan* [tiab] OR prenatal [tiab] OR antenatal [tiab] OR parturition [tiab] OR maternal [tiab] OR gestat* [tiab] OR perinatal [tiab] OR “pre-natal” [tiab] OR “ante natal” [tiab] OR “peri natal” [tiab] OR antepartum [tiab] OR “ante partum” [tiab]) AND ((“Communicable Diseases”[Mesh]) OR “Disease Outbreaks”[Mesh] OR “infectious disease” [tiab:~2] OR “infectious diseases” [tiab:~2] OR “disease outbreak” [tiab:~2] OR “disease outbreaks” [tiab:~2] OR epidemic* [tiab] OR pandemic* [tiab] OR outbreak* [tiab] OR pathogen* [tiab] OR plague* [tiab])) AND (“Clinical Protocols”[Mesh] OR “Clinical Studies as Topic”[Mesh] OR “Epidemiologic Studies”[Mesh] OR “Research Design”[Mesh] OR “Biomedical Research”[Mesh] OR “Data Collection”[Mesh] OR surveillance [tiab] OR “data collection” [tiab] OR trial* [tiab] OR random* [tiab])) AND (“Patient Selection”[Mesh] OR “Eligibility Determination”[Mesh] OR “Research Subjects”[Mesh] OR “patient selection” [tiab:~2] OR “research subject” [tiab:~2] OR “research subjects” [tiab:~2] OR eligibility [tiab] OR “research participant” [tiab:~2] OR “research participants” [tiab:~2])	340
11/8/2023	Embase	(‘pregnancy’/exp OR ‘pregnancy’ OR pregnant OR ‘prenatal’/exp OR prenatal OR antenatal OR perinatal OR ‘maternal’/exp OR maternal) AND (‘outbreaks’ OR ‘pandemics’/exp OR ‘pandemics’ OR ‘epidemics’/exp OR ‘epidemics’) AND (((‘clinical’/exp OR clinical) AND protocols OR ‘clinical’/exp OR clinical) AND (‘studies’/exp OR studies) OR ‘research’/exp OR research) AND (((‘patient’/exp OR patient) AND (‘selection’/exp OR selection) OR ‘research’/exp OR research) OR ‘surveillance’ AND subjects OR ‘eligibility criteria’/exp OR ‘eligibility criteria’)	180
11/8/2023	CINAHL	(‘pregnancy’/exp OR ‘pregnancy’ OR pregnant OR ‘prenatal’/exp OR prenatal OR antenatal OR perinatal OR ‘maternal’/exp OR maternal) AND (‘outbreaks’ OR ‘pandemics’/exp OR ‘pandemics’ OR ‘epidemics’/exp OR ‘epidemics’) AND (((‘clinical’/exp OR clinical) AND protocols OR ‘clinical’/exp OR clinical) AND (‘studies’/exp OR studies) OR ‘research’/exp OR research) OR ‘surveillance’ AND (((‘patient’/exp OR patient) AND (‘selection’/exp OR selection) OR ‘research’/exp OR research) AND subjects OR ‘eligibility criteria’/exp OR ‘eligibility criteria’)	121
11/10/2023	Web of Science	((pregnancy or pregnant or prenatal or antenatal or perinatal or maternal) AND (outbreaks or pandemics or epidemics) AND (clinical protocols or clinical studies or research or surveillance) AND (patient selection or research subjects or eligibility criteria))	374
11/10/2023	Scopus	((pregnancy or pregnant or prenatal or antenatal or perinatal or maternal) AND (outbreaks or pandemics or epidemics) AND (clinical protocols or clinical studies or research or surveillance) AND (patient selection or research subjects or eligibility criteria))	27
11/10/2023	Global Health	((pregnancy or pregnant or prenatal or antenatal or perinatal or maternal) and (outbreaks or pandemics or epidemics) and (clinical protocols or clinical studies or research or surveillance) and (patient selection or research subjects or eligibility criteria)).mp. [mp=abstract, title, original title, heading words, cabicodes words]	10
		Total	1,052

The initial search yielded 1,052 publications. An additional four studies from grey literature were identified through citation searching and an internet-based search of relevant health organization websites using the Google search engine with the search phrase “*pregnant women in clinical research during outbreaks*” and applying the selection criteria outlined below. After removing duplicate articles, 807 publication titles and abstracts were manually screened for relevance and applicability to the research question.

### Selection criteria

To be included in this review, studies were required to discuss conducting research on pregnant women during epidemic- and pandemic-prone disease outbreaks as outlined by the World Health Organization (WHO)^(14)^. Articles not available in English, published before 2017, addressing HIV/AIDS, or without abstracts or full-text availability were excluded. This timeframe was selected due to a notable increase in published literature on this topic beginning in 2017. HIV/AIDS was excluded because the extensive volume of literature on the subject exceeded the scope of this review.

**Figure 2 -:**
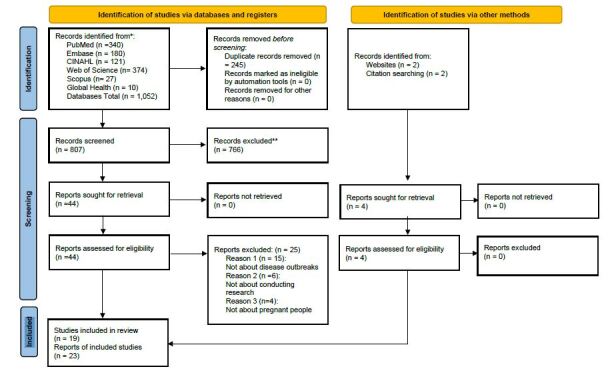
Characterization of articles according to author and year of publication, sample, period and study setting, results, observational measures and level of evidence and quality. United States of America, 2023

Forty-four articles met these criteria and were further reviewed for eligibility, of which 25 were excluded due to irrelevance to the research question or inappropriate study populations. The remaining 23 articles met the eligibility criteria to be included for evaluation and synthesis. The PRISMA-ScR^(15)^ flowchart illustrates the results and literature selection process and is outlined in Figure 2, and findings from the 23 selected articles are charted in Figure 3.

## Results

**Figure 3 –:** PRISMA-ScR flowchart used to identify and select studies. United States of America, 2023

**Article number**	**Author and date**	**Type of study**	**Sample, sample size, setting**	**Relevant findings**	**Observable measures**	**Limitations**	**Evidence level, quality**
1	Al-Shimari, et al., 2023	Systematic review of observational studies	-44 reports published between 2011 and 2022 of hepatitis E outbreaks in 19 countries -Outbreak(s): Hepatitis E globally (2011-2022)	-4 of 44 outbreak reports included pregnancy screening as a method for outbreak detection -68% of the reports noted the number of confirmed cases among pregnant women -91% of the reports did not mention if the vaccine was given to pregnant women to prevent further transmission or future outbreaks	-Characteristics reported in outbreak studies -Interventions to prevent, detect, and contain outbreaks	-Authors noted limited and missing data, as well as gaps in public reporting processes	Level III, Quality B
2	Alirol, et al., 2017	Observational	-WHO-ERC* reviewed 24 protocols and 22 amendments for research studies on Ebola from Aug 2014 to April 2016 -Outbreak(s): Ebola in West Africa (2014-2016)	-Principles of justice and equitable access must be considered -WHO-ERC* requested that protocol amendments include pregnant women unless exclusion was justified by data demonstrating that the risk of treatment was likely to exceed potential benefits. -A global consultation was recommended on the criteria for including pregnant women in interventional studies for high-risk conditions to inform new regulations and guidelines	-Ethical reviews of observational studies and interventional protocols, including vaccinations	-Authors noted protocol inconsistencies, missing information, complex informed consents, as well as the time pressure inherent to accelerated reviews	Level III, Quality B
3	Costantine, et al., 2020	Observational	Online registry search of the international clinical trials database (www.clinicaltrials.gov) using the search term “COVID”, yielding 588 studies -Outbreak(s): COVID-19 global pandemic (2020)	-Only four studies (less than 1%) were specifically designed for pregnant women -More than two-thirds of the interventional studies specifically listed pregnancy as an exclusion criterion. -Missed opportunities to collect pregnancy-specific safety and efficacy data	-Studies specifically designed for pregnant women -Interventional studies -Studies with pregnancy as exclusion/inclusion criteria	-Study limited to trials available in a single registry	Level III, Quality B
4	Edwards & Kochhar, 2020	Expert opinion	-Ethics of conducting clinical research in an outbreak setting -Outbreak(s): Ebola in West Africa (2014-2016); Ebola in the DRC ^†^ (2018-2020)	-Pregnant women were excluded from vaccine trials and denied the Ebola vaccine in West Africa due to insufficient evidence of vaccine safety -Early community engagement, careful articulation of study design and risks and benefits of participation, and post-trial access to the intervention for participating populations, if deemed safe and effective, are recommended. -Impact of these recommendations on the DRC ^†^ Ebola outbreak in 2020	-Ethical considerations, core requirements, and recommendations for enhancing clinical trials during outbreaks	-Potential for author bias	Level V, Quality B
5	Einav, et al., 2020	Observational	Online registry search of the international clinical trials database (www.clinicaltrials.gov) using the search terms “COVID,” or “coronavirus,” or “SARS-CoV-2” up to April 2020, yielding 371 interventional studies -Outbreak(s): COVID-19 global pandemic (2020)	-68% of studies listed pregnancy as an exclusion criterion -76% of trials testing drugs excluded pregnant subjects -31% avoid mention of pregnancy at all in inclusion or exclusion criteria -Several trials investigating drugs with known safety profiles in pregnancy, interventions already used in pregnancy, or low-risk non-pharmacological interventions excluded pregnant women -Lack of differentiation of risk between stages of pregnancy -The assumed unwillingness of pregnant women to participate was a commonly cited reason for exclusion	-Interventional studies with pregnancy as an exclusion/inclusion criterion	-Study limited to trials available in single registry	Level III, Quality B
6	Ethics Working Group on ZIKV Research and Pregnancy, 2017	Consensus panel	-Working group of 15 interdisciplinary experts; consultations with over 60 experts -Outbreak(s): Zika in the Americas (2016)	-Prioritize the development of vaccines acceptable for use in pregnancy during outbreaks -Timely collection of data about vaccine safety and efficacy during pregnancy -Pregnant women should have fair access to vaccine trials with potential for benefit	-Guidance for Zika vaccine research among pregnant women -3 imperatives and 15 recommendations	-Potential for author bias	Level IV, Quality B
7	Farrell, et al., 2020	Expert opinion	-Ethical frameworks for inclusion in clinical trials -Outbreak(s): COVID-19 global pandemic (2020)	-Significant research gaps exist in COVID-19 surveillance among pregnant women -Pregnant women were excluded from drug trials, including hydroxychloroquine, remdesivir, and antiretrovirals -Recommends presumptive inclusion of pregnant women, not requiring consent from any other person, and prioritizing preclinical data needed for inclusion in future trials	-Outlines ethical frameworks and recommended strategies for inclusion in research	-Potential for author bias	Level V, Quality B
8	Frey, et al., 2019	Expert opinion	-Description of ongoing surveillance efforts to monitor Zika virus infections during pregnancy in the U.S. -Outbreak(s): Zika in the Americas (2016)	-Documentation and timely reporting of pregnancy status as part of laboratory testing and case reporting, link this with infant outcomes data - A surveillance system allows for more rapid response to the next public health emergency	-Existing surveillance systems -Strategies for future improvement	-Potential for author bias	Level V, Quality B
9	Goldfarb, et al., 2018	Qualitative	-Pregnant and recently postpartum women attending prenatal care at a large U.S. hospital completed surveys ( *n* =128) -Outbreak(s): Zika (hypothetical)	-77% accepted participation in at least one trial, more likely to accept if inactivated virus -Main motivators for participation included existing evidence of safety (59%), the desire to protect oneself (65%) and one’s baby (98%), perceptions of vaccine safety, and provider recommendations.	-Willingness of participants to enroll in a hypothetical vaccine trial -Attitudes and motivations for participation in vaccine research	-Relatively homogeneous sample -Limited geographic representation in high-income setting	Level III, Quality B
10	Gomes, et al., 2017	Observational	-Review of pregnancy-related eligibility criteria of 16 therapeutic and vaccine studies in Ebola-affected countries -Outbreak(s): Ebola in West Africa (2014-2016)	-All 13 studied drug and vaccine trials excluded pregnant women -Two out of three convalescent plasma studies included pregnant women -Information on pregnancy status was not routinely collected	-Drug and vaccine trials – proposed, initiated or completed-- and their pregnancy-related eligibility criteria	-Potential for limited and missing data	Level III, Quality B
11	Gould, et al., 2021	Observational	-Review of research modifications implemented at a research institute in Australia conducting research on pregnant women -Outbreak(s): COVID-19 global pandemic (2020)	-Online recruitment of pregnant subjects, electronic consent, virtual interviews, distribution of study products by mail, use of personal protective equipment, shorter interviews, increased hygiene, and COVID screening for in-person meetings	-Modified practices for safely conducting perinatal research during the COVID-19 pandemic	-Authors note that technologies used for remote research are in the early stages of adoption -Recruitment and data collection strategies may influence participant characteristics	Level III, Quality B
12	Jaffe, et al., 2020	Qualitative	-Semi-structured in-depth interviews with pregnant and recently pregnant women (n = 13) in the U.S. -Outbreak(s): Zika (hypothetical)	-Participation depended upon three main factors: existing evidence, risk of harm to the baby, and trust of their provider and the medical establishment in general	-Perspectives on participation in hypothetical vaccine trials -Decision-making processes around participation	-Authors note a small, homogeneous sample and brief interviews	Level III, Quality B
13	Kissler, et al., 2022	Observational	-Review of research modifications implemented by 5 academic centers conducting perinatal research in the United States from Mar-May 2020 -Outbreak(s): COVID-19 global pandemic (2020)	-Online recruitment, virtual enrollment and consent, qualitative data collection via video conferencing, use of smartphone technology, wearable biological measurement, and participant self-collection of samples -Increased access to underrepresented populations	-Modified practices to safely conduct perinatal research during the COVID-19 pandemic	-Limited geographic representation in a single high-income setting	Level III, Quality B
14	Knight, et al., 2020	Expert opinion	-Advocacy paper addressing safety concerns and changing attitudes toward inclusion -Outbreak(s): COVID-19 global pandemic (2020)	-Researchers must step out of their comfort zone to change the default -The RECOVERY trial provides a template for the inclusion of pregnant women in other studies	-Safety concerns, changing attitudes	-Potential for author bias	Level V, Quality B
15	Kons, et al., 2022	Observational	-Online registry search of international clinical trials database (www.clinicaltrials.gov) using the search terms “COVID-19 and vaccine” from May-Oct 2020, yielding 90 vaccine trials and 495 treatment trials - Outbreak(s): COVID-19 global pandemic (2020)	- Of 90 vaccine trials, 88 (97.8%) excluded pregnant individuals - Of the 495 treatment trials, 350 (70.7%) excluded pregnant individuals -25% of trials required contraception	-Rates of exclusion for pregnant women in vaccine and treatment trials -Requirements for contraception in pregnancy-capable participants	-Study limited to trials available in single registry	Level III, Quality B
16	Malhamé, et al., 2020	Expert opinion	-Advocacy paper about the moral imperative to include pregnant women in clinical trials -Outbreak(s): COVID-19 global pandemic (2020)	-Moral obligation to protect women through research, rather than from research	-Need to adapt current clinical trials to include pregnant women	-Potential for author bias	Level V, Quality B
17	Marban-Castro, et al., 2021	Qualitative	-Semi-structured interviews with 24 pregnant women and 6 healthcare providers of pregnant women in Spain from June-Oct 2020 -Outbreak(s): COVID-19 global pandemic (2020)	-Willingness to participate in a clinical trial among pregnant women was low (16.7%), and did not depend on which drug was being tested -Fears of adverse effects on the fetus was the principal concern -More likely to participate in trials if a personal safety benefit was perceived	-Participants’ understanding of and potential enrollment in COVID-19 clinical trials	-Authors noted the potential for desirability bias -Small sample size restricted to a single geographic region	Level III, Quality B
18	Minchin, et al., 2023	Systematic review of observational studies	-Vaccine phase II and III clinical trials during outbreaks from 2009-2019, registry search of 18 clinical trial registries globally, yielding 84 results -Outbreak(s): H1N1 influenza, Middle East Respiratory Syndrome Coronavirus, Zika, and Ebola	-Pregnant women were excluded from >80.0 % of emergency vaccine clinical trials -8 clinical trial protocols explicitly included pregnant women -Less than 3% of studies monitored and reported incidental pregnancies -Addresses gaps in existing guidance for inclusion of pregnant women	-Eligibility criteria -Pregnancy outcomes -Gaps in existing guidance	-Limited to studies written in English -Authors noted limitations and gaps in available data	Level III, Quality B
19	Mourad, et al., 2020	Expert opinion	-Advocacy paper for the inclusion of pregnant women in COVID-19 research -Outbreak(s): COVID-19 global pandemic (2020)	-42 of 2100 clinical trials during COVID-19 are being conducted in pregnancy -Less than 2% of current COVID-19 trials include pregnant women -Recommend flexibility in approval processes, collaboration, allowing virtual consent	-Impact of COVID-19 on ongoing and new pregnancy research -Challenges, key strategies necessary	-Potential for author bias	Level V, Quality B
20	The Pregnancy Research Ethics for Vaccines, Epidemics, and New Technologies (Prevent Working Group, 2021)	Consensus panel	-Recommendations from the Prevent Working Group -Outbreak(s): emerging epidemic threats globally	-Stronger surveillance systems with relevant maternal health data -Research should include pregnant women, be developed ahead of outbreaks, include specific recommendations for use in pregnancy, with pregnancy-specific adverse effects -Suitability in pregnancy should guide vaccine investment decisions -Informed consent for participation, including during outbreaks, consider perspectives of pregnant women and maternal health experts in vaccine development	-22 key recommendations for incorporating the interests of pregnant women in vaccine research for emerging pathogens	-Potential for author bias	Level IV, Quality B
21	Smith, et al., 2020	Observational	-Online registry search of the WHO ^§^ International Clinical trials registry platform (18 registries) for COVID-19-related trials in April 2020, yielding 927 results -Outbreak(s): COVID-19 global pandemic (2020)	-52% explicitly excluded pregnancy, 46% failed to address pregnancy at all -16 trials were pregnancy-specific, only 3 were randomized controlled trials -Only 2 drug trials included pregnant women -688 (74.2%) of COVID-19 trials were in Asia, 128 (13.8%) in Europe, and 66 (7.2%) in North America.	-Eligibility criteria for the inclusion or exclusion of pregnant women, categorized by region	-Authors noted that other relevant trials my not have been captured in this analysis -Lack of consistent search functions across databases	Level III, Quality B
22	Whitehead & Walker, 2020	Expert opinion	-Advocacy paper for the inclusion of pregnant women in COVID-19 research -Outbreak(s): COVID-19 global pandemic (2020)	-Advocacy groups and professional organizations must press for the safe inclusion of pregnant women in clinical trials for COVID-19 therapeutics and vaccines	-Historical and ethical arguments for inclusion	-Potential for author bias	Level V, Quality B
23	World Health Organization, 2020	Consensus panel	-Guidelines for the management of pregnant and breastfeeding women in the context of Ebola virus disease, Feb 2020 -Outbreak(s): Ebola virus globally	-Recommends that pregnant women be included in clinical trial vaccine research with provisions for safety monitoring and case documentation, with follow-up of pregnant women and their offspring -Recommends offering the use of investigational therapies for pregnant women with Ebola in the context of rigorous research and following the MEURI ^‡^ protocol	-Key recommendations for the management of pregnant women in the context of Ebola	-Authors noted the very low quality of existing evidence	Level IV, Quality B

*WHO-ERC = World Health Organization Ethics Review Committee; ^†^DRC = Democratic Republic of the Congo; ^‡^MEURI = Monitored Emergency Use of Unregistered and Investigational Interventions; ^§^WHO = World Health Organization

This review included 23 studies published between 2017 and 2023. The Johns Hopkins Nursing Evidence-Based Practice Model was used to evaluate the level and quality of evidence for each article^(16)^. The levels of evidence ranged from III to V, with all studies rated as “good” or assigned a B-quality rating.

Two of the included studies were systematic reviews of observational studies^(8,17)^. Three studies utilized a qualitative methodology, with one using surveys^(18)^ and two employing semi-structured interviews^(19-20)^. Eight studies were observational, five of which involved online searches of clinical trial registry databases^(2,17,21-23)^. Three studies were consensus panels from expert working groups and health organizations^(3,24-25)^, and the remaining seven articles were classified as expert opinions, including advocacy papers and ethical frameworks.

The studies were further classified by disease pathogen, with 12 studies focusing on SARS-CoV-2 (COVID-19), four studies discussing Ebola virus disease (EVD), four studies discussing Zika virus (ZIKV), two studies on multiple pathogens or emerging epidemic threats more broadly, and one study discussing hepatitis E. Seven studies reviewed data from the United States, three from West Africa, two from Australia, one from the United Kingdom, one from the Democratic Republic of the Congo (DRC), one from Spain, and the remaining eight studies analyzed data from multiple or global sources.

Upon review of these studies, six major themes emerged:

### Exclusion from research during outbreaks

There was a consensus in the reviewed literature that pregnant women have been largely excluded from research during outbreaks and pandemics globally. Across regions and throughout multiple disease outbreaks, several studies found that pregnancy was specifically listed as an exclusion criterion for research studies^(2,4,17,21-23,26-28)^, or that a negative pregnancy test was required for participation^(2,17,22)^. Commonly cited reasons for exclusion included concerns about fetal safety, insufficient evidence on the safety of the intervention during pregnancy, assumed unwillingness of pregnant women to participate, or the categorization of pregnant women as a vulnerable population at a greater risk of adverse effects from interventions than non-pregnant adults^(2,7,18,21)^.

Across studies searching clinical trial registry databases, rates of exclusion of pregnant women were between 52% and 97.8%^(2,18,21-23)^. Pregnant women were more likely to be denied eligibility for vaccine development research^(17,22,26)^ and trials testing pharmaceutical treatments^(4,21,23,26)^. During the EVD outbreak in West Africa (2014-2016), pregnant women were excluded from nearly all therapeutic and vaccine trials^(2,6-7,17,25-26)^. This categorical disqualification persisted during the COVID-19 pandemic^(2,4,21-22,27)^. Pregnant women were excluded from investigations of COVID-19 drugs with known safety profiles in pregnancy, interventions already used regularly in pregnancy, and low-risk non-pharmacological interventions^(4-5,21)^. Few clinical trials conducted during COVID-19 were specifically designed for pregnant women, and very few of these were randomized controlled trials^(2,23)^.

### Ethical and moral concerns

There was also a consensus in the reviewed literature that the automatic disqualification of pregnant women from participation in research during outbreaks and pandemics constitutes an ethical and moral issue. Several studies cited violations of ethical principles, including justice, equitable access, informed consent, and autonomy^(2,6,24,28-29)^. Excluding pregnant women under the classification of a vulnerable population in need of protection from exploitation denies them the potential benefits available to other trial participants and fails to respect their capacity to autonomously weigh the risks and benefits of participation and provide informed consent^(2,6)^.

### Calls for a shift to presumptive inclusion

The literature consistently recommended a shift toward the presumptive inclusion of pregnant women in research during outbreaks and pandemics^(2-6)^. Pregnant women should only be disqualified from participation in clinical research if there is a clear justification for exclusion supported by data demonstrating that the risks of treatment are likely to exceed the benefits for the mother, the fetus, or both^(2,4,6)^. Voluntary enrollment in research should be based solely on the participant’s informed consent and not require additional consent from others^(3-4)^. The literature emphasized the importance of presumptive inclusion of pregnant women in trials evaluating therapeutics with known safety profiles in pregnancy and low-risk non-pharmacological interventions^(2,21)^.

### Varied willingness to participate in research

Pregnant women’s willingness to participate in research during disease outbreaks and pandemics varied across the reviewed literature. The proportion willing to enroll in clinical trials ranged from 16.7% for a COVID-19 treatment trial^(20)^ to 77% for a hypothetical Zika virus vaccine trial^(18-19)^. The desire to protect their baby from harm was cited as the strongest motivator for both trial participation and non-participation^(18-19)^. Women were more likely to participate in clinical trials if there was existing safety evidence from prior research, particularly studies on pregnant women, highlighting the importance of collecting data and accurately communicating pregnancy-specific outcomes^(18-20)^. Trial participation decisions were more heavily influenced by recommendations from healthcare providers and public health authorities than those from family, friends, or the government^(18-19)^. The reviewed literature emphasized that the perspectives of pregnant women are essential considerations when designing and implementing clinical research involving pregnant participants^(3,7,19-21)^.

### Importance of pregnancy-specific data collection

Another key theme in the literature was the importance of pregnancy-specific data collection in the context of research during disease outbreaks and pandemics. Many authors highlighted data gaps and missed opportunities to obtain safety and efficacy data for pregnant women during outbreaks^(2-4,8,17,24,26,30)^. Data from non-pregnant individuals cannot be automatically generalized to pregnant women due to the physiological changes of pregnancy. Yet, information on pregnancy status and outcomes is not routinely collected or is often insufficient for long-term monitoring^(2,4,17,24,26,30)^. The lack of standardized data surveillance and reporting creates barriers to effective outbreak prevention and response efforts^(3,8,24,30)^. The literature recommends strengthening coordinated data surveillance systems and routinely collecting maternal health data to support the inclusion of pregnant women in future research and to inform public health responses during outbreaks^(3-4,8,30)^.

### Calls for global institutional and strategic changes

The literature consistently called for a global transformation of research strategies implemented to protect the health of pregnant women during outbreaks^(3,5-7,25,27-28,31-32)^. It was widely acknowledged that shifting toward presumptive inclusion will require changing attitudes, fostering new multidisciplinary collaborations, and reconsidering legal and regulatory research frameworks^(3,5,7)^. Clinical researchers, decision-making bodies, and research funders were urged to prioritize the development of therapeutics and vaccines that are suitable for use in pregnancy, including alternative options for those contraindicated, as well as the collection of preclinical data and pregnancy-specific indicators^(3-4,7)^. Clinical researchers were encouraged to implement effective modifications to research practices during outbreaks to ensure the safe continuation of maternal health research^(27,31-32)^. Finally, advocacy groups, advisory bodies, and professional organizations were urged to push for the inclusion of pregnant women in trials for therapeutics and vaccinations for emerging pathogens^(3,25,28)^.

## Discussion

Conducting research involving pregnant women is ethically and logistically complex. It involves collaboration among multiple stakeholders, including national policymakers, research institutions, regulatory authorities, research funders and sponsors, vaccine and pharmaceutical manufacturers, and oversight bodies. Current international research regulations are intricate, and concerns about legal liability influence eligibility decisions. Existing regulations from the Council of International Organizations of Medical Sciences (CIOMS), the Pan American Health Organization (PAHO), and Subpart B of the United States Code of Federal Regulations (45 CFR Part 46, also known as the “Common Rule”) clearly affirm that pregnant women should be regarded as competent adults capable of providing informed consent for participation in trials that offer potential benefits for them^(3,33-34)^. Additionally, CIOMS and the Federal Policy for the Protection of Human Subjects updated their policies in 2016 and 2019, respectively, to acknowledge that pregnancy alone does not classify an individual as part of a “vulnerable population” in the context of research participation^(3)^.

The literature cited some important examples of the successful inclusion of pregnant women in clinical trials during outbreaks, which can inform future guidelines and research practice. In 2019, the WHO Strategic Advisory Group of Experts (SAGE) recommended that clinical trials for Ebola virus vaccines in the DRC include pregnant and breastfeeding women, with close monitoring for adverse effects until the birth of their infants^(7)^. Four months later, the National Ethics Committee at the School of Public Health at the University of Kinshasa approved the administration of this vaccine to pregnant women after their first trimester^(7)^. However, more than ten months elapsed from the start of the 2020 Ebola outbreak in the DRC for the vaccine to be administered to pregnant women^(7)^.

Another example is the Randomized Evaluation of COVID-19 Therapy (RECOVERY) trial in the United Kingdom in 2020, which tested treatments for patients hospitalized with COVID-19 pneumonia and included pregnant and breastfeeding women^(35)^. The study design leveraged existing data on lopinavir-ritonavir for HIV treatment during pregnancy and hydroxychloroquine for lupus treatment during pregnancy^(5)^. The researchers also adapted some interventions to make them safe for pregnant participants, such as substituting dexamethasone with prednisolone or hydrocortisone to avoid adverse fetal effects^(5)^. Regulatory barriers were addressed by incorporating safety reviews from maternal health experts. One author described this study as “an example of success in equity for pregnant women”^(5)^.

a) Based on the critical evaluation and synthesis of the evidence in the literature, the following summary recommendations have been developed for conducting clinical research involving pregnant women during outbreaks and pandemics globally. Note that these recommendations apply to all pregnant individuals regardless of gender identity.
b) Pregnant women should only be excluded from clinical research during outbreaks if clear, data-driven justification demonstrates that the proposed risks of an intervention likely exceed the expected benefits.
c) Ethical review committees and relevant professional organizations should request protocol amendments and advocate for decision reversals when pregnant women are excluded from clinical trials during outbreaks without clear justification.
d) The potential risks and benefits of participation in clinical trials during outbreaks should be clearly communicated to pregnant participants. Participation decisions should be based on the principle of informed consent and should not require approval from any other party.
e) Standardized data collection systems that include pregnancy-specific indicators should be developed, strengthened, and implemented for the timely collection of data during disease outbreaks.
f) Community engagement, patient education, and communication of existing evidence to potential pregnant participants by trusted health providers should be prioritized before and during clinical trials in the context of a disease outbreak.
g) The development of interventions deemed acceptable for use in pregnancy during outbreaks should be prioritized, and financial incentives should align with this priority.
h) Data from preclinical trials necessary for the future inclusion of pregnant women in clinical trials during an outbreak should be prioritized.
i) Researchers should consult maternal health experts regarding the inclusion or exclusion of pregnant women in clinical trials during outbreaks.
j) The perspectives of pregnant women should be considered when designing and implementing clinical trials in which pregnant women may enroll.

Researchers should implement evidence-based modifications to research protocols to ensure the safe continuation of perinatal research during disease outbreaks.

This paper contributes to advancing scientific knowledge by highlighting the systemic exclusion of pregnant women from research during disease outbreaks and identifying ethical, logistical, and methodological barriers to their inclusion. It synthesizes key findings from the literature to advocate for a paradigm shift toward the presumptive inclusion of pregnant women in clinical research. Finally, it proposes evidence-based recommendations to inform future strategies for improving maternal and fetal health outcomes during public health emergencies.

This literature review aims to synthesize available research on pregnant women during outbreaks and pandemics globally. While the findings provide valuable insight on the topic, the studies reviewed had important limitations. Most of the evidence came from high-income countries, limiting generalizability to middle- and low-income countries. The levels of evidence were relatively low, with no experimental or quasi-experimental studies identified. These findings highlight the need for high-quality research utilizing experimental designs addressing this topic. Additionally, most articles focused on a single disease outbreak in a specific geographical region, which may not be generalizable to all disease outbreaks and pandemics globally.

## Conclusion

This scoping review highlights the systemic exclusion of pregnant women from clinical research during disease outbreaks, revealing significant ethical concerns, knowledge gaps, and missed opportunities to advance maternal and fetal health. Key knowledge gaps identified include the lack of standardized pregnancy-specific data collection, limited research on the safety and efficacy of interventions during pregnancy, and the scarcity of studies from low- and middle-income countries. Through the synthesis of 23 studies across multiple outbreaks, this review identifies barriers such as inconsistent inclusion criteria and inadequate preclinical data, which hinder evidence-based clinical decision-making. By synthesizing existing evidence, forming actionable recommendations, and identifying knowledge gaps, this review provides a foundation for future efforts to advance maternal health research during public health emergencies.
