# Supplementary material for: The inclusion of pregnant women in research during disease outbreaks globally: A scoping review
Source: Rev Lat Am Enfermagem. 2025 Jul 11;33:e4599. doi: 10.1590/1518-8345.7517.4599 (PMC12259220; doi:10.1590/1518-8345.7517.4599)
Supplement: Translation

# Translation: Inclusão de mulheres grávidas em pesquisas durante surtos de doenças em todo o mundo: revisão de escopo

**DOI:** 10.1590/1518-8345.7517.4600

**Authors:** Haley Kartchner Williams, Maria Docal, Angela Chang Chiu, Catherine Ling, Nancy R Reynolds

**Affiliations:** 1 Johns Hopkins University, School of Nursing, Baltimore, Maryland, Estados Unidos da América

**Keywords:** Mulheres Grávidas, Gravidez, Surtos de Doenças, Pesquisa Biomédica, Estudos Clínicos como Assunto, Determinação da Elegibilidade

## Abstract

esta revisão de escopo tem como objetivo mapear a literatura sobre a condução de pesquisas com mulheres grávidas durante surtos globais e sintetizar as evidências para identificar temas, analisar lacunas de conhecimento e fazer recomendações baseadas em evidências para orientar pesquisas futuras.

foi realizada uma busca abrangente da literatura em novembro de 2023 nos bancos de dados *PubMed*, Embase, CINAHL, *Web of Science*, *Scopus* e *Global Health*. Os termos-chave incluíram “pregnancy”, “disease outbreaks”, “biomedical research” e “eligibility criteria”. Vinte e três artigos publicados entre 2017 e 2023 foram incluídos na síntese.

esta revisão identificou um padrão de exclusão de mulheres grávidas em pesquisas conduzidas durante surtos de doenças em diferentes regiões. As taxas de exclusão variaram de 52% a 97,8%. Questões éticas e morais foram levantadas, enfatizando a necessidade de mudanças para que a inclusão de mulheres grávidas em pesquisas seja a regra. Foram propostas recomendações para discutir os riscos e benefícios da participação dessa população em pesquisas, estimular mudanças institucionais e estratégicas globais e padronizar a coleta de dados específicos durante a gravidez, a fim de orientar ações de saúde pública.

esta revisão de escopo destaca a exclusão sistêmica de mulheres grávidas da pesquisa científica durante surtos de doenças, ressaltando preocupações éticas, lacunas críticas de conhecimento e barreiras, ao mesmo tempo em que fornece uma base para o avanço da pesquisa em saúde materna durante emergências de saúde pública.

## Destaques

**(1)** Mulheres grávidas têm sido excluídas de pesquisas durante surtos em nível global. **(2)** A inelegibilidade automática de mulheres grávidas viola princípios éticos. **(3)** A literatura recomenda mudanças para que a inclusão de mulheres grávidas em pesquisas torne-se a regra. **(4)** Os sistemas de vigilância que coletam dados específicos sobre a gravidez devem ser fortalecidos. **(5)** São necessárias mudanças institucionais e estratégicas globais para priorizar a inclusão de mulheres grávidas.

## Introdução

Mulheres grávidas representam uma subpopulação relevante para pesquisas, com 140 milhões de mulheres no mundo dando à luz a cada ano ^( 1 )^. No entanto, mulheres grávidas em todo o mundo são sistemicamente excluídas de pesquisas biomédicas. Esse padrão de “proteção por exclusão” tem restringido a participação de mulheres grávidas e até mesmo de mulheres em idade fértil em ensaios clínicos, no desenvolvimento de medicamentos e em ensaios clínicos para o desenvolvimento de vacinas ^( 2 )^. O motivo mais citado para essa exclusão é a proteção do feto ^( 2 )^. Embora os riscos potenciais de conduzir pesquisas envolvendo mulheres grávidas devam ser cuidadosamente considerados, a exclusão automática impede que a mãe e o bebê se beneficiem potencialmente de uma intervenção e levanta preocupações éticas relevantes ^( 2 )^.

Na década de 1990, relatórios importantes destacaram a sub-representação de mulheres grávidas na agenda de pesquisa, levando a mudanças políticas e legislativas significativas ^( 3 )^. No entanto, apesar dos esforços crescentes para garantir sua inclusão, essas recomendações não foram amplamente implementadas, e as mulheres grávidas continuam a ser significativamente sub-representadas como participantes de ensaios, particularmente em ensaios intervencionistas e de medicamentos ^( 4 - 5 )^. Portanto, a maioria dos tratamentos atuais para condições médicas nunca foi estudada na gravidez, levando os médicos a prescreverem tratamentos e medicamentos “*off-label*”, ou seja, sem ensaios clínicos rigorosos ou evidências que apoiem de forma suficiente sua segurança e eficácia em mulheres grávidas ^( 2 , 6 )^.

Surtos de doenças e pandemias são momentos cruciais para a realização de pesquisas clínicas, pois frequentemente representam as únicas ocasiões em que há um número suficiente de pacientes infectados para viabilizar ensaios clínicos de vacinas e tratamentos ^( 7 )^. É importante destacar que mulheres grávidas apresentam maior risco de desenvolver formas graves de doenças e, em geral, enfrentam taxas mais elevadas de mortalidade e morbidade em comparação com a população geral em diversos surtos, incluindo a doença pelo vírus Ebola (DVE), o vírus Zika, a hepatite E, a febre de Lassa e a influenza ^( 7 - 8 )^. O DVE é possivelmente o exemplo mais marcante, com a mortalidade materna relacionada ao Ebola estimada em aproximadamente 90% e taxas de perdas fetais e neonatais próximas de 100% ^( 6 )^. O vírus Zika pode ser transmitido da mãe para o feto, resultando em restrição do crescimento, anomalias cerebrais fetais e perda fetal ^( 9 )^.

Portanto, é fundamental obter uma perspectiva global sobre essa questão, pois o risco de futuras pandemias é maior do que nunca, representando uma ameaça iminente à segurança sanitária global ^( 10 )^. Aproximadamente 70% dos profissionais de saúde e socorristas em todo o mundo podem engravidar, e sua inclusão em pesquisas durante surtos é essencial para orientar as respostas de saúde pública ^( 11 )^. Os desafios da saúde pública são ainda mais acentuados em países de baixa e média renda (PBMRs), pois estes podem não dispor de recursos adequados para responder a surtos e atender mulheres que vivem em contextos humanitários, como campos de refugiados e zonas de conflito.

Esta revisão de escopo foi conduzida seguindo a estrutura do *Joanna Briggs Institute* (JBI) para revisões de escopo ^( 12 )^ e tem como objetivo mapear a literatura sobre a condução de pesquisas com mulheres grávidas durante surtos em nível global, sintetizar as evidências para identificar temas, investigar lacunas de conhecimento e fazer recomendações baseadas em evidências para orientar pesquisas futuras. O público-alvo inclui profissionais de saúde, pesquisadores, formuladores de políticas e organizações globais de saúde responsáveis pela saúde materno-infantil. Esta revisão é necessária para abordar questões éticas e a escassez de dados específicos sobre a gravidez, o que leva os médicos a se basearem em suposições não baseadas em evidências, comprometendo os desfechos de saúde materna e fetal ^( 2 , 6 )^. A realização desta revisão se justifica por fornecer recomendações passíveis de implementação para promover a equidade e melhorar os desfechos de saúde durante emergências sanitárias.

## Métodos

### Desenho do estudo

Esse estudo é uma revisão de escopo e não recebeu financiamento externo. Para garantir fidelidade e rigor, os principais componentes da estrutura do JBI para revisões de escopo foram utilizados como base metodológica ^( 12 )^, e o relatório seguiu as diretrizes *Preferred Reporting Items for Systematic Reviews and Meta-Analyses Extension for Scoping Reviews* (PRISMA-ScR) ^( 13 )^. A estratégia de busca foi orientada pela questão de pesquisa: “como as mulheres grávidas no mundo todo foram incluídas na pesquisa durante surtos de doenças e pandemias?” Uma busca abrangente da literatura foi conduzida por uma equipe de três revisores em novembro de 2023, em Baltimore, Maryland, EUA, nos bancos de dados eletrônicos *PubMed*, Embase, CINAHL, *Web of Science*, *Scopus* e *Global Health*, utilizando os seguintes descritores: ‘pregnancy’ OR ‘maternal’ AND ‘disease outbreaks’ OR ‘epidemics’ OR ‘pandemics’ AND ‘clinical studies’ OR ‘research’ OR ‘surveillance’ AND ‘patient selection’ OR ‘research subjects’ OR ‘eligibility criteria’. Uma estratégia de busca detalhada, com palavras-chave MeSH e termos associados, é apresentada na Figura 1. A equipe utilizou o *Covidence* como ferramenta de gerenciamento de citações para agilizar o processo de revisão, removendo registros duplicados, atribuindo estudos a revisores individuais e organizando os critérios para a seleção e avaliação dos estudos.

**Figura 1 –:** Termos de pesquisa

**Data da pesquisa**	**Mecanismo de busca**	**Termos de pesquisa**	**Citações recuperadas**
07/11/2023	*PubMed*	(((((“Pregnancy”[Mesh]) OR “Obstetrics”[Mesh]) OR “Pregnant Women”[Mesh] OR pregnan* [tiab] OR prenatal [tiab] OR antenatal [tiab] OR parturition [tiab] OR maternal [tiab] OR gestat* [tiab] OR perinatal [tiab] OR “pre-natal” [tiab] OR “ante natal” [tiab] OR “peri natal” [tiab] OR antepartum [tiab] OR “ante partum” [tiab]) AND ((“Communicable Diseases”[Mesh]) OR “Disease Outbreaks”[Mesh] OR “infectious disease” [tiab:~2] OR “infectious diseases” [tiab:~2] OR “disease outbreak” [tiab:~2] OR “disease outbreaks” [tiab:~2] OR epidemic* [tiab] OR pandemic* [tiab] OR outbreak* [tiab] OR pathogen* [tiab] OR plague* [tiab])) AND (“Clinical Protocols”[Mesh] OR “Clinical Studies as Topic”[Mesh] OR “Epidemiologic Studies”[Mesh] OR “Research Design”[Mesh] OR “Biomedical Research”[Mesh] OR “Data Collection”[Mesh] OR surveillance [tiab] OR “data collection” [tiab] OR trial* [tiab] OR random* [tiab])) AND (“Patient Selection”[Mesh] OR “Eligibility Determination”[Mesh] OR “Research Subjects”[Mesh] OR “patient selection” [tiab:~2] OR “research subject” [tiab:~2] OR “research subjects” [tiab:~2] OR eligibility [tiab] OR “research participant” [tiab:~2] OR “research participants” [tiab:~2])	340
08/11/2023	Embase	(‘pregnancy’/exp OR ‘pregnancy’ OR pregnant OR ‘prenatal’/exp OR prenatal OR antenatal OR perinatal OR ‘maternal’/exp OR maternal) AND (‘outbreaks’ OR ‘pandemics’/exp OR ‘pandemics’ OR ‘epidemics’/exp OR ‘epidemics’) AND (((‘clinical’/exp OR clinical) AND protocols OR ‘clinical’/exp OR clinical) AND (‘studies’/exp OR studies) OR ‘research’/exp OR research) AND (((‘patient’/exp OR patient) AND (‘selection’/exp OR selection) OR ‘research’/exp OR research) OR ‘surveillance’ AND subjects OR ‘eligibility criteria’/exp OR ‘eligibility criteria’)	180
08/11/2023	CINAHL	(‘pregnancy’/exp OR ‘pregnancy’ OR pregnant OR ‘prenatal’/exp OR prenatal OR antenatal OR perinatal OR ‘maternal’/exp OR maternal) AND (‘outbreaks’ OR ‘pandemics’/exp OR ‘pandemics’ OR ‘epidemics’/exp OR ‘epidemics’) AND (((‘clinical’/exp OR clinical) AND protocols OR ‘clinical’/exp OR clinical) AND (‘studies’/exp OR studies) OR ‘research’/exp OR research) OR ‘surveillance’ AND (((‘patient’/exp OR patient) AND (‘selection’/exp OR selection) OR ‘research’/exp OR research) AND subjects OR ‘eligibility criteria’/exp OR ‘eligibility criteria’)	121
10/11/2023	*Web of Science*	((pregnancy or pregnant or prenatal or antenatal or perinatal or maternal) AND (outbreaks or pandemics or epidemics) AND (clinical protocols or clinical studies or research or surveillance) AND (patient selection or research subjects or eligibility criteria))	374
10/11/2023	*Scopus*	((pregnancy or pregnant or prenatal or antenatal or perinatal or maternal) AND (outbreaks or pandemics or epidemics) AND (clinical protocols or clinical studies or research or surveillance) AND (patient selection or research subjects or eligibility criteria))	27
10/11/2023	*Global Health*	((pregnancy or pregnant or prenatal or antenatal or perinatal or maternal) and (outbreaks or pandemics or epidemics) and (clinical protocols or clinical studies or research or surveillance) and (patient selection or research subjects or eligibility criteria)).mp. [mp=abstract, title, original title, heading words, cabicodes words]	10
		Total	1.052

A busca inicial resultou em 1.052 publicações. Quatro estudos adicionais da literatura cinzenta foram identificados por meio da busca de citações e de uma busca na internet em sites relevantes de organizações de saúde, utilizando o mecanismo de busca *Google* com a frase “*pregnant women in clinical research during outbreaks*” [mulheres grávidas em pesquisa clínica durante surtos] e aplicando os critérios de seleção descritos abaixo. Após a remoção de artigos duplicados, 807 títulos e resumos foram analisados manualmente para verificar sua relevância e aplicabilidade à questão de pesquisa.

### Critérios de seleção

Para serem incluídos nesta revisão, os estudos deveriam discutir a condução de pesquisas sobre mulheres grávidas durante surtos de doenças propensas a epidemias e pandemias, conforme descrito pela *World Health Organization* (WHO) ^( 14 )^. Artigos não disponíveis em inglês, publicados antes de 2017, que abordassem HIV/AIDS ou que não possuíssem resumos ou o texto completo disponível, foram excluídos. Esse período foi selecionado devido a um aumento notável no volume de artigos publicados sobre o tema a partir de 2017. HIV/AIDS foi excluído devido ao extenso volumede literatura sobre o tópico, que excedeu o escopo desta revisão. 

Quarenta e quatro artigos atenderam a esses critérios e foram revisados posteriormente para elegibilidade, dos quais 25 foram excluídos por não serem relevantes para a questão de pesquisa ou por abordarem outras populações. Vinte e três artigos atenderam aos critérios de elegibilidade e foram incluídos para avaliação e síntese. O fluxograma PRISMA-ScR ^( 15 )^ detalha os resultados e o processo de seleção da literatura e está apresentado na Figura 2. Os resultados dos 23 artigos selecionados são apresentados na Figura 3.

## Resultados

**Figura 2 –:** Fluxograma PRISMA-ScR utilizado para identificação e seleção dos estudos. Estados Unidos da América, 2023

**Figura 3 –:** Descrição dos artigos, segundo autor e ano, tipo de estudo, amostra, período e cenário, resultados, medidas observacionais, limitações, nível de evidencia e qualidade. Estados Unidos da América, 2023

**Número do artigo**	**Autor e data**	**Tipo de estudo**	**Amostra, período e cenário do estudo**	**Resultados relevantes**	**Medidas observáveis**	**Limitações**	**Nível de evidência, qualidade**
1	Al-Shimari, et al., 2023	Revisão sistemática de estudos observacionais	-44 Artigos publicados entre 2011-2022 sobre surtos de hepatite E em 19 países - Surto(s): Hepatite E em nível global (2011-2022)	-4 de 44 relatórios de surto incluíram triagem de gravidez como um método de detecção de surto -68% dos relatórios observaram o número de casos confirmados entre mulheres grávidas -91% dos relatórios não mencionaram se a vacina foi administrada a mulheres grávidas para prevenir transmissão posterior ou surtos futuros	- Características incluídas nos relatórios de surtos -Intervenções para prevenir, detectar e conter surtos	- Os autores mencionaram dados limitados e ausentes, bem como lacunas nos processos de relatórios públicos.	Nível III, Qualidade B
2	Alirol, et al., 2017	Observacional	-WHO-ERC* revisou 24 protocolos e 22 emendas para estudos de pesquisa sobre o Ebola de agosto de 2014 a abril de 2016 - Surto(s): Ebola na África Ocidental (2014-2016)	-- Princípios de justiça e acesso equitativo devem ser considerados - A WHO-ERC* solicitou alterações no protocolo para incluir mulheres grávidas, a menos que a exclusão fosse justificada por dados que demonstrassem que o risco do tratamento provavelmente excederia os riscos - Recomendou uma consulta global sobre os critérios para inclusão de mulheres grávidas em estudos intervencionais para condições de alto risco para orientar novos regulamentos e diretrizes	- Revisões éticas para estudos observacionais e protocolos intervencionistas, incluindo vacinação	- Os autores notaram inconsistências no protocolo, informações ausentes, consentimentos informados complexos, além da pressão de tempo inerente às revisões aceleradas	Nível III, Qualidade B
3	Costantine, et al., 2020	Observacional	Pesquisa de registro on-line de banco de dados de ensaios clínicos internacionais (www.clinicaltrials.gov) usando o termo de busca “COVID”, resultando em 588 estudos - Surto(s): pandemia global de COVID-19 (2020)	- Apenas quatro estudos (menos de 1%) foram projetados especificamente para mulheres grávidas - Mais de dois terços dos estudos intervencionais adicionaram especificamente a gravidez como critério de exclusão -Oportunidades perdidas de coletar dados de segurança e eficácia específicos da gravidez	-Estudos projetados especificamente para mulheres grávidas -Estudos intervencionais -Estudos com a gravidez como critério de exclusão/inclusão	-Estudo limitado a ensaios disponíveis em registro único	Nível III, Qualidade B
4	Edwards & Kochhar, 2020	Opinião de especialista	- Ética na condução de pesquisas clínicas em um cenário de surto - Surto(s): Ebola na África Ocidental (2014-2016); Ebola na RDC ^†^ (2018-2020)	- Mulheres grávidas foram excluídas dos testes de vacinas e de receber a vacina contra o Ebola na África Ocidental devido a evidências insuficientes de segurança da vacina - Recomenda o envolvimento precoce da comunidade, e a explicitação clara do desenho do estudo, riscos e benefícios da participação e acesso pós-teste à intervenção para populações participantes se forem consideradas seguras e eficazes - Impacto dessas recomendações durante o surto de Ebola na RDC ^†^ em 2020	-Considerações éticas, requisitos essenciais e recomendações para melhorar os ensaios clínicos durante surtos	-Potencial de viés do autor	Nível V, Qualidade B
5	Einav, et al., 2020	Observacional	Busca de registro *online* de banco de dados de ensaios clínicos internacionais (www.clinicaltrials.gov) usando os termos de busca “COVID ou coronavírus ou SARS-COV-2” até abril de 2020, resultando em 371 estudos intervencionais - Surto(s): pandemia global de COVID-19 (2020)	-68% dos estudos listaram a gravidez como um critério de exclusão -76% dos ensaios que testam medicamentos excluíram gestantes -31% evitam mencionar a gravidez em critérios de inclusão ou exclusão -Vários ensaios que investigaram medicamentos com perfis de segurança conhecidos na gravidez, intervenções já usadas na gravidez ou intervenções não farmacológicas de baixo risco excluíram gestantes -Falta de diferenciação de risco entre os estágios da gravidez -A suposta falta de vontade das gestantes em participar foi o motivo comumente citado para a exclusão	-Estudos intervencionistas com a gravidez como critério de exclusão/inclusão	-Estudo limitado a ensaios disponíveis em registro único	Nível III, Qualidade B
6	*Ethics Working Group on ZIKV Research and Pregnancy* , 2017	Painel de consenso	-Grupo de trabalho de 15 especialistas interdisciplinares; consultas com mais de 60 especialistas -Surto(s): Zika nas Américas (2016)	-Priorizar o desenvolvimento de vacinas seguras para uso na gravidez durante surtos -Coleta oportuna de dados sobre a segurança e eficácia da vacina durante a gravidez -Mulheres grávidas devem ter acesso justo a testes de vacinas com potencial de benefício	-Orientação para pesquisa da vacina contra o Zika entre mulheres grávidas -3 imperativos e 15 recomendações	-Potencial de viés do autor	Nível IV, Qualidade B
7	Farrell, et al., 2020	Opinião de especialista	Estruturas éticas para inclusão em ensaios clínicos - Surto(s): pandemia global de COVID-19 (2020)	- Existem lacunas significativas de pesquisa na vigilância da COVID-19 entre mulheres grávidas - Mulheres grávidas foram excluídas de ensaios de medicamentos, incluindo hidroxicloroquina, remdesivir e antirretrovirais - Recomenda que a inclusão de mulheres grávidas se torne padrão, não exigindo consentimento de nenhuma outra pessoa e priorizando dados pré-clínicos necessários para inclusão em ensaios futuros	-Descreve estruturas éticas e estratégias recomendadas para inclusão em pesquisas	-Potencial de viés do autor	Nível V, Qualidade B
8	Frey, et al., 2019	Opinião de especialista	--Descrição dos esforços de vigilância em andamento para monitorar a infecção pelo vírus Zika durante a gravidez nos EUA -Surto(s): Zika nos EUA (2016)	- Documentação e relatórios oportunos do estado da gravidez como parte dos testes laboratoriais e relatórios de casos, vinculando aos dados de resultados infantis - Um sistema de vigilância permite uma resposta mais rápida à próxima emergência de saúde pública	- Sistemas de vigilância existentes -Estratégias para melhorias futuras	-Potencial de viés do autor	Nível V, Qualidade B
9	Goldfarb, et al., 2018	Qualitativo	- Mulheres grávidas e puérperas que recebiam cuidados pré-natais em um grande hospital dos EUA responderam às pesquisas, n=128 - Surto(s): Zika (hipotético)	-77% aceitaram participar de pelo menos um ensaio, e eram mais propensas a aceitar se o vírus fosse inativado -Os principais motivadores para a participação incluíram evidências existentes de segurança (59%), desejo de proteger a si mesma (65%) e ao bebê (98%), percepções de segurança da vacina e recomendação do profissional de saúde	- Vontade de participar de um ensaio hipotético de vacina - Atitudes e motivações para participação em pesquisa de vacina	-Amostra relativamente homogênea -Representação geográfica limitada a um ambiente de alta renda	Nível III, Qualidade B
10	Gomes, et al., 2017	Observacional	- Revisão dos critérios de elegibilidade relacionados à gravidez de 16 estudos terapêuticos e de vacinas em países afetados pelo Ebola - Surto(s): Ebola na África Ocidental (2014-2016)	-Todos os 13 ensaios de medicamentos e vacinas estudados excluíram mulheres grávidas -Dois dos três estudos de plasma convalescente incluíram mulheres grávidas -Informações sobre o estado de gravidez não foram coletadas rotineiramente	- Ensaios clínicos de medicamentos e vacinas propostos, iniciados ou concluídos e critérios de elegibilidade relacionados à gravidez	-Potencial para dados limitados e ausentes	Nível III, Qualidade B
11	Gould, et al., 2021	Observacional	-Revisão de modificações de pesquisa implementadas em um instituto de pesquisa na Austrália conduzindo pesquisa em mulheres grávidas -Surto(s): pandemia global de COVID-19 (2020)	Recrutamento *online* de gestantes, consentimento eletrônico, entrevistas virtuais, envio de produtos de estudo por correio e uso de equipamento de proteção individual, entrevistas mais curtas, maior higiene e triagem de COVID para reuniões presenciais	- Práticas modificadas para conduzir com segurança pesquisas perinatais durante a pandemia de COVID-19	- Os autores observam que as tecnologias usadas para pesquisa remota estão em estágios iniciais de uso - As estratégias de recrutamento e captura de dados podem impactar as características dos participantes	Nível III, Qualidade B
12	Jaffe, et al., 2020	Qualitativo	-Entrevistas semiestruturadas em profundidade com mulheres grávidas e recentemente grávidas (n = 13) nos EUA -Surto(s): Zika (hipotético)	- A participação dependia de três fatores principais: evidências existentes, risco de danos ao bebê e confiança no serviço/profissional de saúde e no sistema médico em geral	-Perspectivas sobre a participação em ensaios clínicos hipotéticos de vacinas -Processos de tomada de decisão participativos	- Os autores observam uma amostra pequena e homogênea e entrevistas de curta duração	Nível III, Qualidade B
13	Kissler, et al., 2022	Observacional	-Revisão de modificações de pesquisa implementadas por 5 centros acadêmicos que conduzem pesquisa perinatal nos Estados Unidos, de março a maio de 2020 -Surto(s): pandemia global de COVID-19 (2020)	- Recrutamento *online* , inscrição e consentimento virtuais, coleta de dados qualitativos por meio de videoconferência, uso de tecnologia de *smartphone* , uso de dispositivo biológico vestível para monitoramento e auto coleta de amostras pelos participantes - Maior acesso a populações sub-representadas	- Práticas modificadas para conduzir com segurança pesquisas perinatais durante a pandemia de COVID-19	-Representação geográfica em um ambiente de alta renda	Nível III, Qualidade B
14	Knight, et al., 2020	Opinião de especialista	Artigo defendendo a abordagem de preocupações relacionadas a segurança e mudança de atitudes em relação à inclusão - Surto(s): pandemia global de COVID-19 (2020)	- Os pesquisadores devem sair de sua zona de conforto para mudar o padrão - O teste *Recovery* fornece um modelo para inclusão de mulheres grávidas em outros estudos	- Preocupações com a segurança e mudança de atitudes	-Potencial de viés do autor	Nível V, Qualidade B
15	Kons, et al., 2022	Observacional	- Busca de registro on-line de banco de dados de ensaios clínicos internacionais (www.clinicaltrials.gov) usando os termos de busca “COVID-19 e vacina” de maio a outubro de 2020, resultando em 90 ensaios de vacinas e 495 ensaios de tratamento -Surto(s): pandemia global de COVID-19	- 88 (97,8%) dos 90 ensaios de vacinas excluíram gestantes - Dos 495 ensaios de tratamento, 350 (70,7%) excluíram gestantes - 25% dos ensaios exigiram contracepção	- Taxas de exclusão para mulheres grávidas em ensaios de vacinas e tratamentos - Requisitos para contracepção em participantes férteis	-Estudo limitado a ensaios disponíveis em registro único	Nível III, Qualidade B
16	Malhamé, et al., 2020	Opinião de especialista	-Artigo advogando a necessidade moral de incluir mulheres grávidas em ensaios clínicos -Surto(s): pandemia global de COVID-19 (2020)	- Obrigação moral de proteger as mulheres por meio de pesquisas ao invés de impedi-las de participar das pesquisas.	-Necessidade de adaptar os ensaios clínicos atuais para incluir mulheres grávidas	-Potencial de viés do autor	Nível V, Qualidade B
17	Marban-Castro, et al., 2021	Qualitativo	-Entrevistas semiestruturadas com 24 mulheres grávidas e 6 profissionais de saúde de mulheres grávidas na Espanha de junho a outubro de 2020 -Surto(s): pandemia global de COVID-19 (2020)	- A disposição das mulheres grávidas para participar de um ensaio clínico foi baixa (16,7%) e não dependeu de qual medicamento estava sendo testado - O medo de efeitos adversos no feto foi a principal preocupação - Mais propensos a participar de ensaios se o benefício de segurança pessoal fosse percebido	- Compreensão das participantes e potencial participação em ensaios clínicos da COVID-19	- Os autores observaram potencial viés de desejabilidade social - Tamanho pequeno da amostra, limitado a uma região geográfica	Nível III, Qualidade B
18	Minchin, et al., 2023	Revisão sistemática de estudos observacionais	-- Ensaios clínicos de vacinas em fases II e III durante os surtos de 2009-2019, busca de registro de 18 registros de ensaios clínicos globalmente, produzindo 84 resultados - Surto(s): influenza H1N1, Coronavírus da Síndrome Respiratória do Oriente Médio, Zika e Ebola	-Mulheres grávidas foram excluídas de >80,0% dos ensaios clínicos de vacinas de emergência -8 protocolos de ensaios clínicos incluíram explicitamente mulheres grávidas -Menos de 3% dos estudos monitoraram e relataram gestações não planejadas -Aborda lacunas nas orientações existentes para inclusão de mulheres grávidas	-Critérios de elegibilidade -Resultados da gravidez -Lacunas nas diretrizes existentes	-Limitado a estudos em inglês -Autores observaram limitações e lacunas nos dados	Nível III, Qualidade B
19	Mourad, et al., 2020	Opinião de especialista	-Artigo advogando a inclusão de mulheres grávidas em pesquisas sobre COVID-19 -Surto(s): pandemia global de COVID-19 (2020)	-42 dos 2100 ensaios clínicos durante a COVID-19 foram conduzidos durante a gravidez -Menos de 2% dos ensaios atuais da COVID-19 incluem mulheres grávidas -Recomenda flexibilidade nos processos de aprovação, colaboração, permitindo consentimento virtual	-Impacto da COVID-19 na pesquisa em andamento e nova sobre gravidez -Desafios e estratégias-chave necessárias	-Potencial de viés do autor	Nível V, Qualidade B
20	*The Pregnancy Research Ethics for Vaccines, Epidemics, and New Technologies* ( *Prevent Working Group* , 2021)	Painel de consenso	--Recomendações do *Prevent Working Group* -Surto(s): ameaças epidêmicas emergentes globais	- Sistemas de vigilância mais robustos com dados relevantes sobre saúde materna - A pesquisa deve incluir mulheres grávidas, ser desenvolvida antes dos surtos e conter recomendações específicas para uso na gravidez, incluindo efeitos adversos específicos. - A adequação na gravidez deve orientar as decisões de investimento em vacinas - Consentimento informado para participação, inclusive durante os surtos, considerar as perspectivas de mulheres grávidas e especialistas em saúde materna no desenvolvimento de vacinas	-22 recomendações-chave para a inclusão dos interesses das mulheres grávidas nas vacinas contra patógenos emergentes	-Potencial de viés do autor	Nível IV, Qualidade B
21	Smith, et al., 2020	Observacional	-Pesquisa de registro on-line da plataforma de registro de ensaios clínicos internacionais da WHO ^§^ (18 registros) para ensaios relacionados à COVID-19 em abril de 2020, gerando 927 resultados -Surto(s): pandemia global da COVID-19 (2020)	-52% excluíram explicitamente a gravidez, 46% não abordaram a gravidez de forma alguma -16 ensaios foram específicos para gravidez, apenas 3 foram ensaios clínicos randomizados -Apenas 2 ensaios de medicamentos incluíram mulheres grávidas -688 (74,2%) dos ensaios de COVID-19 aconteceram na Ásia, 128 (13,8%) na Europa e 66 (7,2%) na América do Norte.	-Critérios de elegibilidade para inclusão/exclusão de gestantes, categorizados por região	- Os autores reconhecem que outros ensaios clínicos relevantes podem não ter sido capturados pela análise - Inconsistência nas funções de pesquisa entre os bancos de dados	Nível III, Qualidade B
22	Whitehead & Walker, 2020	Opinião de especialista	- Artigo advogando a inclusão de mulheres grávidas na pesquisa sobre COVID-19 - Surto(s): pandemia global de COVID-19 (2020)	- Grupos de defesa e organizações profissionais devem defender a inclusão segura de mulheres grávidas em ensaios clínicos para terapias e vacinas contra a COVID-19	- Argumentos históricos e éticos para inclusão	-Potencial de viés do autor	Nível V, Qualidade B
23	*World Health Organization* , 2020	Painel de consenso	- Diretrizes para o manejo de mulheres grávidas e lactantes no contexto da doença do vírus Ebola, fevereiro 2020 - Surto(s): vírus Ebola globalmente	-Recomenda que mulheres grávidas sejam incluídas em pesquisas de vacinas de ensaios clínicos com disposições para monitoramento de segurança e documentação de casos, com acompanhamento de mulheres grávidas e seus descendentes -Recomenda oferecer o uso de terapias investigacionais para mulheres grávidas com Ebola no contexto de pesquisas rigorosas e de acordo com o protocolo MEURI ^‡^	- Principais recomendações para o tratamento de mulheres grávidas no contexto do Ebola	- Os autores observam uma qualidade muito baixa das evidências existentes	Nível IV, Qualidade B

*WHO-ERC = Comitê de Revisão de Ética da Organização Mundial da Saúde; ^†^RDC = República Democrática do Congo; ^‡^MEURI = *Monitored Emergency Use of Unregistered and Investigational Interventions*; ^§^WHO = *World Health Organization*

Foram incluídos nesta revisão 23 estudos publicados entre 2017 e 2023. O modelo *Johns Hopkins Nursing Evidence-Based Practice* foi utilizado para avaliar o nível e a qualidade da evidência de cada artigo ^( 16 )^. Os níveis de evidência variaram de III a V, com todos os estudos considerados “bons” ou com classificação de qualidade B.

Dois dos estudos incluídos são revisões sistemáticas de estudos observacionais ^( 8 , 17 )^. Três estudos utilizaram uma abordagem qualitativa, sendo um baseado em pesquisas ^( 18 )^ e dois em entrevistas semiestruturadas ^( 19 - 20 )^. Oito estudos são observacionais, com cinco deles envolvendo buscas *online* em bancos de dados de registros de ensaios clínicos ^( 2 , 17 , 21 - 23 )^. Três estudos são painéis de consenso de grupos de trabalho de especialistas e organizações de saúde ^( 3 , 24 - 25 )^, e os sete artigos restantes foram classificados como opiniões de especialistas, incluindo artigos de *advocacy* e estruturas éticas.

Os estudos foram classificados de acordo com o patógeno da doença, com 12 estudos abordando o SARS-CoV-2 (COVID-19), quatro a doença pelo vírus Ebola (DVE), quatro o vírus Zika, dois estudos abordando múltiplos patógenos ou ameaças epidêmicas emergentes em geral e um a hepatite E. Sete estudos analisam dados dos Estados Unidos, três da África Ocidental, dois da Austrália, um do Reino Unido, um da República Democrática do Congo (RDC), um da Espanha e os oito estudos restantes discutem dados de fontes múltiplas ou globais.

Seis temas principais emergiram após a revisão dos estudos.

### Exclusão de pesquisas durantes surtos

Houve um consenso nos estudos incluídos nesta revisão de que mulheres grávidas foram amplamente excluídas de pesquisas em todo o mundo durante surtos e pandemias. Em diversas regiões e em diferentes surtos de doenças, vários artigos identificaram que a gravidez foi listada especificamente como critério de exclusão em estudos de pesquisa ^( 2 , 4 , 17 , 21 - 23 , 26 - 28 )^ ou que um teste de gravidez negativo era exigido para a participação ^( 2 , 17 , 22 )^. Os motivos comumente citados para exclusão incluíram preocupações com a segurança fetal, evidências insuficientes sobre a segurança da intervenção na gravidez, a suposta falta de vontade das mulheres grávidas em participar ou a categorização de mulheres grávidas como população vulnerável, sob maior risco de efeitos adversos das intervenções do que pessoas adultas não grávidas ^( 2 , 7 , 18 , 21 )^.

As taxas de exclusão de mulheres grávidas variaram entre 52% e 97,8% em todos os estudos que analisaram bancos de dados de registros de ensaios clínicos ^( 2 , 18 , 21 - 23 )^. Mulheres grávidas tinham maior probabilidade de ter a elegibilidade negada para pesquisas de desenvolvimento de vacinas ^( 17 , 22 , 26 )^ e ensaios clínicos que testavam tratamentos farmacêuticos ^( 4 , 21 , 23 , 26 )^. Durante o surto de DVE na África Ocidental entre 2014 e 2016, mulheres grávidas foram excluídas de quase todos os ensaios terapêuticos e de vacinas ^( 2 , 6 - 7 , 17 , 25 - 26 )^. A desqualificação categórica continuou durante a pandemia de COVID-19 ^( 2 , 4 , 21 - 22 , 27 )^. Mulheres grávidas foram excluídas de investigações sobre medicamentos para COVID-19 com perfis de segurança já conhecidos na gravidez, intervenções regularmente utilizadas na gestação ou intervenções não farmacológicas de baixo risco ^( 4 - 5 , 21 )^. Poucos ensaios clínicos conduzidos durante a COVID-19 foram projetados especificamente para mulheres grávidas e somente alguns desses eram ensaios clínicos randomizados ^( 2 , 23 )^.

### Implicações éticas e morais

Também houve um consenso nos estudos incluídos nesta revisão de que a desqualificação automática de mulheres grávidas da participação em pesquisas durante surtos e pandemias constitui um problema ético e moral. Vários estudos citaram violações de princípios éticos, incluindo justiça, acesso equitativo, consentimento informado e autonomia ^( 2 , 6 , 24 , 28 - 29 )^. Negar a elegibilidade de mulheres grávidas sob a justificativa de serem uma população vulnerável que necessita de proteção contra exploração as impede de acessar o benefício potencial oferecido a outros participantes de ensaios clínicos e não respeita sua capacidade de avaliar autonomamente os riscos e benefícios da participação e fornecer consentimento informado ^( 2 , 6 )^.

### Apelos por uma mudança para a inclusão como regra

Houve uma recomendação consistente na literatura para uma mudança para a inclusão presumida, ou seja, a inclusão como regra de mulheres grávidas em pesquisas durante surtos e pandemias ^( 2 - 6 )^. Mulheres grávidas só devem ser desqualificadas da participação em pesquisas clínicas se houver uma justificativa clara para a exclusão, com dados demonstrando que o risco do tratamento provavelmente excederá o benefício para a mãe, o feto ou ambos ^( 2 , 4 , 6 )^. A inscrição voluntária em pesquisas deve ser baseada apenas no consentimento informado da participante e não exigir consentimento adicional de outras pessoas ^( 3 - 4 )^. A literatura enfatizou a importância de que a inclusão de mulheres grávidas em pesquisas que testam intervenções terapêuticas com perfis de segurança conhecidos na gravidez e intervenções não farmacológicas de baixo risco seja a regra ^( 2 , 21 )^.

### Níveis de disposição variados para participar em pesquisas

A disposição de mulheres grávidas em participar de pesquisas durante surtos de doenças e pandemias variou na literatura revisada. A proporção disposta a se inscrever em ensaios clínicos variou de 16,7% para um ensaio clínico de tratamento da COVID-19 ^( 20 )^ a 77% para um ensaio hipotético de vacina contra o vírus Zika ^( 18 - 19 )^. O desejo de proteger o bebê de possíveis prejuízos foi citado como o principal motivador tanto para a participação quanto para a não participação no ensaio ^( 18 - 19 )^. As mulheres eram mais propensas a participar de ensaios clínicos se houvesse evidências de segurança provenientes de pesquisas anteriores, especialmente aquelas envolvendo mulheres grávidas, destacando a importância de coletar dados e comunicar com precisão os desfechos específicos durante a gravidez ^( 18 - 20 )^. As decisões de participação em ensaios clínicos foram mais fortemente influenciadas pelas recomendações do profissional de saúde e da autoridade de saúde pública do que por aquelas de familiares, amigos ou do governo ^( 18 - 19 )^. Os estudos reconheceram que as perspectivas das mulheres grávidas são considerações essenciais ao se projetar e implementar pesquisas clínicas que envolvam participantes grávidas ^( 3 , 7 , 19 - 21 )^.

### Importância da coleta de dados específicos durante a gravidez

Outro tema que surgiu na literatura foi a importância da coleta de dados específicos sobre a gravidez no contexto de pesquisas cientificas durante surtos de doenças e pandemias. Muitos autores observaram lacunas de dados e oportunidades perdidas para obter informações sobre segurança e eficácia para mulheres grávidas durante surtos ^( 2 - 4 , 8 , 17 , 24 , 26 , 30 )^. Dados de mulheres não grávidas não podem ser automaticamente generalizados para mulheres grávidas devido às mudanças fisiológicas da gestação; no entanto, informações sobre o estado da gravidez não são coletadas rotineiramente ou são frequentemente insuficientes para monitoramento de longo prazo ^( 2 , 4 , 17 , 24 , 26 , 30 )^. A falta de vigilância e de relatórios padronizados de dados cria barreiras para atividades eficazes de prevenção e resposta a surtos ^( 3 , 8 , 24 , 30 )^. A literatura recomenda o fortalecimento de sistemas coordenados de vigilância de dados e a coleta rotineira de dados de saúde materna para apoiar a inclusão de mulheres grávidas em pesquisas futuras e informar as respostas de saúde pública durante surtos ^( 3 - 4 , 8 , 30 )^.

### Apelos para mudanças institucionais e estratégicas globais

Houve um apelo comum em toda a literatura revisada por uma transformação global das estratégias de pesquisa implementadas para proteger a saúde de mulheres grávidas durante surtos ^( 3 , 5 - 7 , 25 , 27 - 28 , 31 - 32 )^. Foi amplamente reconhecido que essa mudança em direção à inclusão como regra exigirá mudanças de atitudes, novas colaborações multidisciplinares e a reconsideração de estruturas legais e regulatórias de pesquisa ^( 3 , 5 , 7 )^. Pesquisadores, órgãos decisórios e agências de fomento foram incentivados a priorizar o desenvolvimento de intervenções terapêuticas e vacinas promissoras para uso na gravidez, incluindo opções alternativas para aquelas contraindicadas na gestação, bem como a coleta de dados pré-clínicos e indicadores específicos na gravidez ^( 3 - 4 , 7 )^. Pesquisadores foram incentivados a implementar modificações eficazes nas práticas de pesquisa durante surtos para garantir a continuação segura dos estudos em saúde materna ^( 27 , 31 - 32 )^. Por fim, grupos de defesa, órgãos consultivos e organizações profissionais foram encarregados de pressionar pela inclusão de mulheres grávidas em ensaios de intervenções terapêuticas e vacinas para patógenos emergentes ^( 3 , 25 , 28 )^.

## Discussão

A realização de pesquisas sobre mulheres grávidas é eticamente e logisticamente complexa e envolve diversas partes interessadas, incluindo os responsáveis por formular políticas nacionais, instituições de pesquisa, autoridades regulatórias, agências de fomento, fabricantes de vacinas e produtos farmacêuticos, e órgãos de fiscalização. Os atuais regulamentos internacionais de pesquisa são complexos, e as preocupações com a responsabilidade legal influenciam as decisões de elegibilidade. Os regulamentos existentes do *Council of International Organizations of Medical Sciences* (CIOMS), da Organização Pan-Americana da Saúde (OPAS) e da Subparte B do Código de Regulamentos Federais dos Estados Unidos (45 CFR Parte 46, também conhecido como “*Common Rule*” [Regra Comum]), apoiam claramente que mulheres grávidas sejam consideradas adultas competentes, capazes de fornecer consentimento informado para a participação em ensaios que lhes ofereçam benefícios potenciais ^( 3 , 33 - 34 )^. Além disso, o CIOMS e a Política Federal para a Proteção de Sujeitos Humanos atualizaram suas diretrizes em 2016 e 2019, respectivamente, para reconhecer que a gravidez, por si só, não qualifica alguém como uma “população vulnerável” no contexto da participação em pesquisas científicas ^( 3 )^.

Os estudos citaram alguns exemplos importantes da inclusão bem-sucedida de mulheres grávidas em ensaios clínicos durante surtos, que podem servir de referência para futuras diretrizes e práticas de pesquisa. Em 2019, o Grupo Consultivo Estratégico de Peritos da OMS (SAGE em inglês) recomendou que os ensaios clínicos para vacinas contra o vírus Ebola na RDC incluíssem mulheres grávidas e lactantes, e que as participantes grávidas fossem monitoradas de perto para possíveis efeitos adversos até o nascimento de seus bebês ^( 7 )^. Quatro meses depois, o Comitê Nacional de Ética da Escola de Saúde Pública da Universidade de Kinshasa aprovou a administração dessa vacina para mulheres grávidas após o primeiro trimestre de gravidez ^( 7 )^. No entanto, levou mais de dez meses desde o início do surto de Ebola de 2020 na RDC para que a vacina fosse administrada a mulheres grávidas ^( 7 )^.

Outro exemplo é o ensaio *Randomized Evaluation of COVID-19 Therapy (RECOVERY)*, realizado no Reino Unido em 2020, que testou tratamentos para pacientes internados com pneumonia por COVID-19 e incluiu mulheres grávidas e lactantes ^( 35 )^. O desenho do estudo utilizou dados existentes sobre o uso de Lopinavir+Ritonavir no tratamento do HIV durante a gravidez e de hidroxicloroquina para o tratamento do lúpus em gestantes ^( 5 )^. Os pesquisadores também adaptaram algumas intervenções para torná-las adequadas ao uso por mulheres grávidas, como substituir a dexametasona por prednisolona ou hidrocortisona para evitar efeitos fetais adversos ^( 5 )^. As barreiras regulatórias foram superadas com revisões de segurança conduzidas por especialistas em saúde materna. Um dos autores descreveu o estudo como “um exemplo de sucesso em equidade para mulheres grávidas” ^( 5 )^.

a) Com base na avaliação crítica e síntese das evidências presentes na literatura, foram resumidas as seguintes recomendações para a condução de pesquisas clínicas em mulheres grávidas durante surtos e pandemias em nível global. Deve-se destacar que essas recomendações se aplicam a todas as pessoas grávidas, independentemente de sua identidade de gênero.
b) Mulheres grávidas só devem ser excluídas da participação em pesquisas clínicas durante surtos se houver uma justificativa clara, baseada em dados que demonstrem que os riscos da intervenção provavelmente excedem os benefícios esperados.
c) Comitês de revisão ética e organizações profissionais relevantes devem solicitar alterações no protocolo e defender a reversão da decisão quando mulheres grávidas forem excluídas de ensaios clínicos durante surtos sem uma justificativa clara.
d) Os possíveis riscos e benefícios potenciais da participação em ensaios clínicos durante surtos devem ser claramente informados às participantes grávidas. As decisões de participação devem se basear no princípio do consentimento informado e não devem exigir consentimento de nenhuma outra parte.
e) Sistemas padronizados de coleta de dados, que incluam indicadores específicos da gravidez para a obtenção oportuna de informações durante surtos de doenças, devem ser desenvolvidos, fortalecidos e utilizados.
f) Profissionais de saúde de confiança devem priorizar o envolvimento da comunidade, a educação dos pacientes e a comunicação das evidências existentes a potenciais participantes grávidas antes e durante os ensaios clínicos no contexto de um surto de doença.
g) O desenvolvimento de intervenções seguras e adequadas para uso na gravidez durante surtos deve ser priorizado, e os incentivos financeiros devem estar alinhados com essa prioridade.
h) Devem ser priorizados os dados de ensaios pré-clínicos necessários para a futura inclusão de mulheres grávidas em ensaios clínicos durante um surto.
i) Pesquisadores devem consultar especialistas em saúde materna ao decidir sobre a inclusão ou exclusão de mulheres grávidas em ensaios clínicos durante surtos.
j) As perspectivas das mulheres grávidas devem ser consideradas ao projetar e implementar ensaios clínicos nos quais sua participação seja possível.

Pesquisadores devem implementar modificações baseadas em evidências nos processos de pesquisa para garantir a continuidade segura dos estudos perinatais durante surtos de doenças.

Este artigo contribui para o avanço do conhecimento científico ao destacar a exclusão sistêmica de mulheres grávidas em pesquisas durante surtos de doenças e identificar barreiras éticas, logísticas e metodológicas à sua inclusão. Além disso, sintetiza as principais descobertas da literatura para defender uma mudança de paradigma em direção à inclusão de mulheres grávidas na pesquisa clínica como regra. Por fim, propõe recomendações baseadas em evidências para orientar estratégias futuras visando a melhoria dos desfechos de saúde materna e fetal durante emergências de saúde pública.

Esta revisão de literatura teve como objetivo sintetizar a literatura disponível sobre a condução de pesquisas com mulheres grávidas durante surtos e pandemias em nível global. Embora as descobertas tenham fornecido insights valiosos sobre o tema, os estudos revisados apresentaram limitações importantes. A maioria das evidências veio de países de alta renda, o que limita a generalização para países de média e baixa renda. Além disso, os níveis de evidência foram relativamente baixos, sem a identificação de estudos experimentais ou quase-experimentais. Essas descobertas ressaltam a necessidade de pesquisas de alta qualidade utilizando delineamentos experimentais para abordar essa questão. Por fim, a maioria dos artigos analisou um único surto de doença em uma região geográfica específica, o que pode não ser generalizado para todos os surtos de doenças e pandemias globalmente.

## Conclusão

Esta revisão de escopo destaca a exclusão sistêmica de mulheres grávidas de pesquisas clínicas durante surtos de doenças, revelando preocupações éticas relevantes, lacunas de conhecimento e oportunidades perdidas que poderiam promover a saúde materna e fetal. As principais lacunas de conhecimento identificadas incluem a falta de coleta padronizada de dados específicos durante a gravidez, a escassez de pesquisas sobre a segurança e eficácia de intervenções durante a gestação e a ausência de estudos em países de baixa e média renda. Por meio da síntese de 23 estudos que investigaram diferentes surtos, essa revisão identifica barreiras como critérios de inclusão inconsistentes e dados pré-clínicos inadequados, que dificultam a tomada de decisões clínicas baseadas em evidências. Ao consolidar as evidências existentes, apresentar recomendações viáveis e identificar lacunas de conhecimento, esta revisão fornece uma base para esforços futuros com o objetivo de fortalecer a pesquisa em saúde materna durante emergências de saúde pública.

# Translation: La inclusión de mujeres embarazadas en investigación realizada durante brotes de enfermedades a nivel mundial: una revisión exploratoria

**DOI:** 10.1590/1518-8345.7517.4598

**Authors:** Haley Kartchner Williams, Maria Docal, Angela Chang Chiu, Catherine Ling, Nancy R Reynolds

**Affiliations:** 1 Johns Hopkins University, School of Nursing, Baltimore, Maryland, Estados Unidos de América

**Keywords:** Mujeres Embarazadas, Embarazo, Brotes de Enfermedades, Investigación Biomédica, Estudios Clínicos como Asunto, Determinación de la Elegibilidad

## Abstract

el alcance de esta revisión tiene como objetivos: buscar en la literatura datos relacionados con investigaciones sobre mujeres embarazadas durante brotes a nivel mundial, sintetizar los hallazgos para identificar temas, analizar brechas de conocimiento y hacer recomendaciones basadas en evidencia para orientar investigaciones futuras.

se realizó una búsqueda bibliográfica exhaustiva en noviembre de 2023, utilizando las bases de datos *PubMed*, Embase, CINAHL, *Web of Science*, *Scopus* y *Global Health*. Los términos clave incluyeron “embarazo”, “brote de enfermedades”, “investigación biomédica” y “criterios de elegibilidad”. Se incluyeron para la síntesis 23 artículos publicados entre 2017 y 2023.

esta revisión encontró un patrón de exclusión de mujeres embarazadas en las investigaciones, durante múltiples brotes de enfermedades, en distintas regiones. Las tasas de exclusión oscilaron entre 52% y 97,8%. Se plantearon preocupaciones éticas y morales, haciendo hincapié en la necesidad de cambiar la inclusión presunta de mujeres embarazadas en las investigaciones. Surgieron recomendaciones para: los debates sobre los riesgos y beneficios de la participación en investigaciones; los cambios institucionales y estratégicos globales y, la recopilación estandarizada de datos específicos del embarazo, para orientar las respuestas de la salud pública.

el alcance de esta revisión destaca la exclusión sistemática de las mujeres embarazadas de las investigaciones, durante los brotes de enfermedades, enfatizando las preocupaciones éticas, las brechas críticas de conocimiento y las barreras, al mismo tiempo que se proporciona una base para avanzar en la investigación de la salud materna, durante las emergencias de salud pública.

## Destacados

**(1)** Las mujeres embarazadas han sido excluidas de las investigaciones durante los brotes a nivel mundial. **(2)** La inelegibilidad automática de las mujeres embarazadas viola principios éticos. **(3)** La literatura recomienda, en las investigaciones, cambiar la inclusión presunta de las mujeres embarazadas. **(4)** Se deben fortalecer los sistemas de vigilancia que recopilan datos específicos del embarazo. **(5)** Es necesario realizar cambios institucionales y estratégicos globales para priorizar a las mujeres embarazadas.

## Introducción

Las mujeres embarazadas son una importante sub-población de investigaciones: 140 millones de mujeres en todo el mundo dan a luz cada año^(1)^. Sin embargo, las mujeres embarazadas tienen una larga historia de ser excluidas sistemáticamente de las investigaciones biomédica a nivel mundial. Este patrón de “protección por exclusión” ha limitado a las mujeres embarazadas e incluso a las mujeres en edad fértil de participar en ensayos terapéuticos clínicos, en el desarrollo de medicamentos y ensayos de desarrollo de vacunas^(2)^. La razón de la exclusión, más citada, es la protección del feto^(2)^. Si bien se deben considerar cuidadosamente los riesgos potenciales de realizar investigaciones que involucren mujeres embarazadas, la inelegibilidad automática niega a la madre y al bebé cualquier beneficio potencial de una intervención y presenta preocupaciones éticas sustanciales^(2)^.

En la década de 1990 importantes informes destacaron la subrepresentación de las mujeres embarazadas en la agenda de investigaciones; esto condujo a la realización de cambios políticos y legislativos importantes^(3)^. Sin embargo, a pesar de estos grandes esfuerzos para garantizar la inclusión, estas recomendaciones no se han implementado ampliamente y las mujeres embarazadas continúan estando significativamente subrepresentadas como participantes en ensayos de investigación, en particular en ensayos intervencionistas y de medicamentos^(4-5)^. Por eso, los tratamientos más actuales para afecciones médicas nunca se han estudiado durante el embarazo, lo que lleva a los médicos a recetar tratamientos y medicamentos “no comprobados”, sin ensayos clínicos rigurosos y de evidencias suficientes para dar respaldo, seguridad y eficacia^(2,6)^.

Los brotes de enfermedades y pandemias son momentos esenciales para realizar investigaciones clínicas, ya que a menudo son los únicos períodos con cantidades suficientes de pacientes infectados para realizar ensayos clínicos de vacunas y tratamientos terapéuticos^(7)^. Es importante destacar que las mujeres embarazadas tienen un mayor riesgo de sufrir una enfermedad más grave y, a menudo, tienen un mayor riesgo de mortalidad y morbilidad, que la población general, por múltiples enfermedades propensas a brotes, entre ellas el virus del Ébola (EVE), el virus de la Zika (ZIKA), la hepatitis E, la fiebre de Lassa y la gripe^(7-8)^. El EVE es quizás el ejemplo más llamativo: se estima que la mortalidad materna relacionada con el ébola es de alrededor del 90% y las pérdidas fetales y neonatales se acercan al 100%^(6)^. El ZIKA puede transmitirse de la madre al feto, provocando restricción del crecimiento, anomalías cerebrales fetales y pérdida fetal^(9)^.

Es fundamental adoptar una perspectiva global sobre esta cuestión, ya que el riesgo de futuras pandemias es mayor que nunca y supone una amenaza inminente para la seguridad sanitaria mundial^(10)^. El 70% de las trabajadoras de la salud y las socorristas en todo el mundo pueden quedar embarazadas; así su inclusión en las investigaciones durante los brotes es esencial para orientar las respuestas de la salud pública^(11)^. Los problemas de salud pública se agravan aún más en los países de ingresos bajos y medios (PIBM), que pueden carecer de recursos para responder a los brotes y alcanzar a las mujeres que viven en entornos humanitarios como los campos de refugiados y las zonas de conflicto.

El alcance de esta revisión se llevó a cabo siguiendo el marco del Instituto Joanna Briggs (JBI) para revisiones de este tipo^(12)^; su objetivo es buscar literatura sobre la realización de investigaciones sobre mujeres embarazadas durante brotes a nivel mundial y sintetizar los hallazgos para identificar temas, analizar lagunas de conocimiento y hacer recomendaciones basadas en evidencia para orientar investigaciones futuras. El público objetivo incluye profesionales de la salud, investigadores, formuladores de políticas y organizaciones de salud globales responsables por la salud materno-infantil. Esta revisión es necesaria para abordar preocupaciones éticas y la falta de datos específicos del embarazo, lo que requiere que los médicos se basen en suposiciones “no verificadas”, poniendo así en peligro los resultados de la salud materna y fetal^(2,6)^. La realización de esta revisión se justifica porque proporciona recomendaciones prácticas para garantizar la equidad y mejorar los resultados de la salud durante las emergencias sanitarias.

## Métodos

### Diseño del estudio

Este estudio es una síntesis sistemática de la evidencia y no recibió financiamiento externo. Para garantizar la fidelidad, el rigor, la revisión y su alcance fue utilizada la base metodológica y los componentes clave del marco JBI^(12)^, con informes guiados por la lista de verificación del *Preferred Reporting Items for Systematic Reviews and Meta-Analyses Extension for Scoping Reviews* (PRISMA-ScR)^(13)^. La estrategia de búsqueda se basó en la siguiente pregunta de investigación: “¿Cómo se han incluido las mujeres embarazadas, en la investigación, durante los brotes de enfermedades y pandemias a nivel mundial?”. Un equipo de tres revisores realizó una búsqueda exhaustiva, no ciega, en la literatura, en noviembre de 2023 en Baltimore, Maryland, EEUU, utilizando: *PubMed*, Embase, CINAHL, *Web of Science*, *Scopus*, y las bases de datos electrónicas *Global Health*; utilizando términos claves como: ‘pregnancy’ OR ‘maternal’ AND ‘disease outbreaks’ OR ‘epidemics’ OR ‘pandemics’ AND ‘clinical studies’ OR ‘research’ OR ‘surveillance’ AND ‘patient selection’ OR ‘research subjects’ OR ‘eligibility criteria’. En la Figura 1 se describe una estrategia de búsqueda detallada que utiliza palabras clave MeSH y términos asociados. El equipo utilizó *Covidence* como herramienta de gestión de citas para agilizar el proceso de revisión, eliminando registros duplicados, asignando estudios a revisores individuales y organizando criterios para la selección y evaluación de estudios.

**Figure 1 -:** Términos de búsqueda

**Fecha de búsqueda**	**Motor de búsqueda**	**Términos de búsqueda**	**Citas obtenidas**
07/11/2023	*PubMed*	(((((“Pregnancy”[Mesh]) OR “Obstetrics”[Mesh]) OR “Pregnant Women”[Mesh] OR pregnan* [tiab] OR prenatal [tiab] OR antenatal [tiab] OR parturition [tiab] OR maternal [tiab] OR gestat* [tiab] OR perinatal [tiab] OR “pre-natal” [tiab] OR “ante natal” [tiab] OR “peri natal” [tiab] OR antepartum [tiab] OR “ante partum” [tiab]) AND ((“Communicable Diseases”[Mesh]) OR “Disease Outbreaks”[Mesh] OR “infectious disease” [tiab:~2] OR “infectious diseases” [tiab:~2] OR “disease outbreak” [tiab:~2] OR “disease outbreaks” [tiab:~2] OR epidemic* [tiab] OR pandemic* [tiab] OR outbreak* [tiab] OR pathogen* [tiab] OR plague* [tiab])) AND (“Clinical Protocols”[Mesh] OR “Clinical Studies as Topic”[Mesh] OR “Epidemiologic Studies”[Mesh] OR “Research Design”[Mesh] OR “Biomedical Research”[Mesh] OR “Data Collection”[Mesh] OR surveillance [tiab] OR “data collection” [tiab] OR trial* [tiab] OR random* [tiab])) AND (“Patient Selection”[Mesh] OR “Eligibility Determination”[Mesh] OR “Research Subjects”[Mesh] OR “patient selection” [tiab:~2] OR “research subject” [tiab:~2] OR “research subjects” [tiab:~2] OR eligibility [tiab] OR “research participant” [tiab:~2] OR “research participants” [tiab:~2])	340
08/11/2023	Embase	(‘pregnancy’/exp OR ‘pregnancy’ OR pregnant OR ‘prenatal’/exp OR prenatal OR antenatal OR perinatal OR ‘maternal’/exp OR maternal) AND (‘outbreaks’ OR ‘pandemics’/exp OR ‘pandemics’ OR ‘epidemics’/exp OR ‘epidemics’) AND (((‘clinical’/exp OR clinical) AND protocols OR ‘clinical’/exp OR clinical) AND (‘studies’/exp OR studies) OR ‘research’/exp OR research) AND (((‘patient’/exp OR patient) AND (‘selection’/exp OR selection) OR ‘research’/exp OR research) OR ‘surveillance’ AND subjects OR ‘eligibility criteria’/exp OR ‘eligibility criteria’)	180
08/11/2023	CINAHL	(‘pregnancy’/exp OR ‘pregnancy’ OR pregnant OR ‘prenatal’/exp OR prenatal OR antenatal OR perinatal OR ‘maternal’/exp OR maternal) AND (‘outbreaks’ OR ‘pandemics’/exp OR ‘pandemics’ OR ‘epidemics’/exp OR ‘epidemics’) AND (((‘clinical’/exp OR clinical) AND protocols OR ‘clinical’/exp OR clinical) AND (‘studies’/exp OR studies) OR ‘research’/exp OR research) OR ‘surveillance’ AND (((‘patient’/exp OR patient) AND (‘selection’/exp OR selection) OR ‘research’/exp OR research) AND subjects OR ‘eligibility criteria’/exp OR ‘eligibility criteria’)	121
10/11/2023	*Web of Science*	((pregnancy or pregnant or prenatal or antenatal or perinatal or maternal) AND (outbreaks or pandemics or epidemics) AND (clinical protocols or clinical studies or research or surveillance) AND (patient selection or research subjects or eligibility criteria))	374
10/11/2023	*Scopus*	((pregnancy or pregnant or prenatal or antenatal or perinatal or maternal) AND (outbreaks or pandemics or epidemics) AND (clinical protocols or clinical studies or research or surveillance) AND (patient selection or research subjects or eligibility criteria))	27
10/11/2023	*Global Health*	((pregnancy or pregnant or prenatal or antenatal or perinatal or maternal) and (outbreaks or pandemics or epidemics) and (clinical protocols or clinical studies or research or surveillance) and (patient selection or research subjects or eligibility criteria)).mp. [mp=abstract, title, original title, heading words, cabicodes words]	10
		Total	1.052

Esta búsqueda inicial arrojó 1.052 publicaciones. Se identificaron cuatro estudios adicionales en la literatura gris, mediante una búsqueda de citas y realizando la búsqueda en sitios web de Internet de organizaciones de salud relevantes, utilizando el motor de búsqueda del Google con la frase de búsqueda “mujeres embarazadas en investigación clínica durante brotes” y aplicando los criterios de selección que se describen a continuación. Después de omitir artículos duplicados, se exploraron manualmente 807 títulos y resúmenes de publicaciones para determinar su relevancia y aplicabilidad a la pregunta práctica.

### Criterios de selección

Para ser incluidos en esta revisión, se requirió que los estudios abordaran la realización de investigaciones sobre mujeres embarazadas durante brotes de enfermedades propensas a epidemias y pandemias, según lo descrito por la Organización Mundial de la Salud (OMS)^(14)^. Se excluyeron los artículos no disponibles en inglés, publicados antes de 2017, que abordaron el HIV/SIDA o que no tuvieran resúmenes o disponibilidad de texto completo. Se seleccionó este período de tiempo debido a un aumento notable en el volumen de literatura publicada sobre este tema, a partir de 2017. Se excluyó el HIV/SIDA debido al amplio volumen de literatura sobre el tema, que excedía el alcance de esta revisión.

Cuarenta y cuatro artículos cumplieron estos criterios y fueron revisados más a fondo para determinar su elegibilidad; veinticinco fueron excluidos debido a su insignificancia delante de la pregunta de investigación o por tratarse de poblaciones inapropiadas. Veintitrés artículos cumplieron los criterios de elegibilidad para ser incluidos para evaluación y síntesis. El diagrama de flujo PRIMSA-ScR^(15)^ ilustra además los resultados y el proceso de selección de literatura; este está descrito en la Figura 2; los hallazgos de los 23 artículos seleccionados se representan en la Figura 3.

## Resultados

**Figura 2 –:** Diagrama de flujo PRISMA-ScR utilizado para identificar y seleccionar estudios. Estados Unidos de América, 2023

**Figura 3 -:** Caracterización de los artículos según autor y año de publicación, muestra, período y escenario de estudio, resultados, medidas observacionales y nivel de evidencia y calidad. Estados Unidos de América, 2023

**Número del artículo**	**Autor y fecha**	**Tipo de estudio**	**Muestra, tamaño, configuración**	**Hallazgos relevantes**	**Medidas observables**	**Limitaciones**	**Evidencia nivel, calidad**
1	Al-Shimari, et al., 2023	Revisión sistemática de estudios observacionales	-44 informes publicados entre 2011-2022 de hepatitis E, brotes en 19 países -Brote(s): Hepatitis E globalmente (2011-2022)	-4 de 44 informes de brotes incluyeron la detección del embarazo como método de detección de brotes - El 68% de los informes indicó el número de casos confirmados entre mujeres embarazadas - El 91% de los informes no mencionó si se administró la vacuna a mujeres embarazadas para prevenir una mayor transmisión o futuros brotes	-Características incluidas en los informes de brotes -Intervenciones para prevenir, detectar y contener brotes	-Los autores señalaron datos limitados y faltantes, así como lagunas en los procesos de presentación de informes públicos.	Nivel III, Calidad B
2	Alirol, et al., 2017	Observacional	-El Centro de Respuesta ante Emergencias de la Organización Mundial de la Salud revisó 24 protocolos y 22 enmiendas para estudios de investigación sobre el Ébola desde agosto de 2014 hasta abril de 2016 -Brote(s): Ébola en África occidental (2014-2016)	-Se deben tener en cuenta los principios de justicia y acceso equitativo -La OMS-ERC* solicitó que se modifiquen los protocolos para incluir a las mujeres embarazadas a menos que la exclusión esté justificada por datos que demuestren que el riesgo del tratamiento, probablemente, exceda los beneficios -Recomendó una consulta mundial sobre los criterios para la inclusión de mujeres embarazadas en estudios de intervención para condiciones de alto riesgo a fin de informar nuevas regulaciones y directrices	-Revisiones éticas de estudios observacionales y protocolos de intervención, incluidas las vacunaciones.	-Los autores señalaron inconsistencias en el protocolo, información faltante, consentimientos informados complejos, así como la presión temporal inherente a las revisiones aceleradas.	Nivel III, Calidad B
3	Costantine, et al., 2020	Observacional	194 / 5,000 Búsqueda en el registro online en la base de datos de ensayos clínicos internacionales (www.clinicaltrials.gov) utilizando el término de búsqueda “COVID”; se obtuvieron 588 estudios -Brote(s): pandemia mundial de COVID-19 (2020)	-Solo cuatro estudios (menos del 1 %) fueron diseñados específicamente para mujeres embarazadas -Más de dos tercios de los estudios de intervención agregaron específicamente el embarazo como criterio de exclusión -Oportunidades perdidas para recopilar datos específicos de seguridad y eficacia del embarazo	-Estudios diseñados específicamente para mujeres embarazadas -Estudios de intervención -Estudios con el embarazo como criterio de exclusión/inclusión	-Estudio limitado a ensayos disponibles en un único registro	Nivel III, Calidad B
4	Edwards & Kochhar, 2020	Opinión de expertos	-Ética de la realización de investigaciones clínicas en un contexto de brote -Brote(s): Ébola en África occidental (2014-2016); Ébola en la DRC ^†^ (2018-2020)	-Las mujeres embarazadas fueron excluidas de los ensayos de vacunas y de recibir la vacuna contra el ébola en África occidental, debido a la falta de evidencia sobre la seguridad de la vacuna -Recomienda: participación temprana de la comunidad, articulación cuidadosa del diseño del estudio, evaluar los riesgos y beneficios de la participación, y el acceso posterior al ensayo a la intervención para las poblaciones que participaron si se determina que es segura y eficaz -Impacto de estas recomendaciones durante el brote de ébola en la República Democrática del Congo ^†^ en 2020	-Consideraciones éticas, requisitos básicos y recomendaciones para mejorar los ensayos clínicos durante los brotes	- Potencial de sesgo del autor/a	Nivel V, Calidad B
5	Einav, et al., 2020	Observacional	Búsqueda online en el registro de la base de datos internacional de ensayos clínicos (www.clinicaltrials.gov) utilizando los términos de búsqueda “COVID o coronavirus o SARS-COV-2” hasta abril de 2020, que arrojó 371 estudios de intervención -Brote(s): pandemia mundial de COVID-19 (2020)	- El 68% de los estudios incluyeron el embarazo como criterio de exclusión - El 76% de los ensayos que probaron medicamentos excluyeron mujeres embarazadas - El 31% evita mencionar el embarazo entre los criterios de inclusión o exclusión - Varios ensayos que investigaron medicamentos con perfiles de seguridad conocidos en el embarazo, intervenciones ya utilizadas en el embarazo o intervenciones no farmacológicas de bajo riesgo, excluyeron mujeres embarazadas - Falta de diferenciación de riesgos entre las etapas del embarazo - La supuesta falta de voluntad de las mujeres embarazadas para participar fue el motivo de exclusión más común	-Estudios de intervención con embarazo como criterio de exclusión/inclusión	-Estudio limitado a ensayos disponibles en un único registro	Nivel III, Calidad B
6	Grupo de trabajo de ética sobre la investigación del virus del Zika y el embarazo, 2017	Panel de consenso	-Grupo de trabajo de 15 expertos interdisciplinarios; consultas con más de 60 expertos -Brote(s): Zika en las Américas (2016)	-Priorizar el desarrollo de vacunas aceptables para uso en el embarazo durante los brotes -Recopilación oportuna de datos sobre la seguridad y eficacia de las vacunas durante el embarazo -Mujeres embarazadas deben tener un acceso justo a los ensayos de vacunas con potencial de beneficio	-Orientaciones para la investigación de la vacuna contra el Zika en mujeres embarazadas -3 imperativas y 15 recomendaciones	- Potencial de sesgo del autor/a	Nivel IV, Calidad B
7	Farrell, et al., 2020	Opinión de expertos	- Marcos éticos para la inclusión en ensayos clínicos - Brote(s): pandemia mundial de COVID-19 (2020)	-Existen importantes lagunas en la investigación sobre la vigilancia de la COVID-19 entre las mujeres embarazadas -Se excluyó a las mujeres embarazadas de los ensayos de medicamentos, incluidos los de hidroxicloroquina, remdesivir y antirretrovirales -Recomienda la inclusión presuntiva de las mujeres embarazadas, sin requerir el consentimiento de ninguna otra persona y priorizando los datos preclínicos necesarios para su inclusión en ensayos futuros	-Describe los marcos éticos y las estrategias recomendadas para su inclusión en la investigación.	-Posibilidad de sesgo del autor/a	Nivel V, Calidad B
8	Frey, et al., 2019	Opinión de expertos	- Descripción de las actividades de vigilancia en curso para controlar la infección por el Zika virus durante el embarazo en los EEUU -Brote(s): Zika en América (2016)	-Documentación y notificación oportuna del estado del embarazo como parte de las pruebas de laboratorio y la notificación de casos, vincular esto con los datos de resultados de los bebés - Un sistema de vigilancia permite una respuesta más rápida ante la próxima emergencia de salud pública	- Sistemas de vigilancia existentes -Estrategias para mejoras futuras	-Posibilidad de sesgo del autor/a	Nivel V, Calidad B
9	Goldfarb, et al., 2018	Cualitativo	-Mujeres embarazadas y en posparto reciente que asisten a atención prenatal en un gran hospital de EEUU completaron encuestas, n=128 -Brote(s): Zika (hipotético)	- El 77 % aceptó participar en por lo menos un ensayo, con mayor probabilidad de aceptar si se trataba de un virus inactivado - Los principales motivadores para la participación incluyeron evidencia existente de seguridad (59 %), deseo de protegerse a sí mismo (65 %) y al bebé (98 %), percepciones de seguridad de la vacuna y recomendación del proveedor	-Disposición de los participantes a participar en un ensayo hipotético de vacunas -Actitudes y motivaciones para participar en la investigación de vacunas	-Muestra relativamente homogénea -Representación geográfica limitada en un entorno de altos ingresos	Nivel III, Calidad B
10	Gomes, et al., 2017	Observacionales	-Revisión de los criterios de elegibilidad relacionados con el embarazo de 16 estudios terapéuticos y de vacunas en países afectados por el Ébola -Brote(s): Ébola en África occidental (2014-2016)	-Los 13 ensayos de medicamentos y vacunas estudiados excluyeron a las mujeres embarazadas -Dos de los tres estudios de plasma convaleciente incluyeron mujeres embarazadas -No se recopiló de forma rutinaria información sobre el estado del embarazo	-Ensayos de medicamentos y vacunas propuestos, iniciados o completados y criterios de elegibilidad relacionados con el embarazo	-Posibilidad de datos limitados y faltantes	Nivel III, Calidad B
11	Gould, et al., 2021	Observacionales	-Revisión de las modificaciones de investigación implementadas en un instituto de investigación en Australia, que realiza investigaciones sobre mujeres embarazadas -Brote(s): pandemia mundial de COVID-19 (2020)	-Reclutamiento online de embarazadas, consentimiento electrónico, entrevistas virtuales, envío de productos del estudio por correo y uso de equipos de protección personal, entrevistas más cortas, mayor higiene y detección de COVID para reuniones en persona.	-Prácticas modificadas para realizar investigaciones perinatales de manera segura durante la pandemia de COVID-19	-Los autores señalan que las tecnologías utilizadas para la investigación a distancia se encuentran en las primeras etapas de uso -Las estrategias de reclutamiento y captura de datos pueden afectar las características de los participantes	Nivel III, Calidad B
12	Jaffe, et al., 2020	Cualitativo	- Entrevistas en profundidad semiestructuradas con embarazadas o que estuvieron embarazadas recientemente (n = 13) en los EEUU. -Brote(s): Zika (hipotético)	- La participación dependía de tres factores principales: la evidencia existente, el riesgo de daño al bebé y la confianza del proveedor y del establecimiento médico en general.	- Perspectivas sobre la participación en ensayos hipotéticos de vacunas -Procesos de toma de decisiones en torno a la participación	- Los autores señalan que la muestra es pequeña y homogénea y que las entrevistas son de corta duración.	Nivel III, Calidad B
13	Kissler, et al., 2022	Observacionales	- Revisión de las modificaciones de investigación implementadas por cinco centros académicos que realizan investigaciones perinatales en los Estados Unidos entre marzo y mayo de 2020 -Brote(s): pandemia mundial de COVID-19 (2020)	-Reclutamiento online, inscripción y consentimiento virtuales, recolección de datos cualitativos mediante videoconferencia, uso de tecnología de teléfonos inteligentes, medición biológica portátil y recolección de muestras por parte de los participantes -Mayor acceso a poblaciones subrepresentadas	-Prácticas modificadas para realizar investigaciones perinatales de manera segura durante la pandemia de COVID-19	-Representación geográfica en un entorno de altos ingresos	Nivel III, Calidad B
14	Knight, et al., 2020	Opinión de expertos	- Documento que se refieren a cómo abordar las preocupaciones en materia de seguridad y cambiar las actitudes hacia la inclusión - Brote(s): pandemia mundial de COVID-19 (2020)	-Los investigadores deben salir de su zona de confort para cambiar la situación predeterminada -El ensayo *Recovery* proporciona un modelo para la inclusión de mujeres embarazadas en otros estudios	-Preocupaciones de seguridad, cambio de actitudes	-Posibilidad de sesgo del autor/a	Nivel V, Calidad B
15	Kons, et al., 2022	Observacionales	-Búsqueda online de registros en la base de datos de ensayos clínicos internacionales (www.clinicaltrials.gov) utilizando los términos de búsqueda “COVID-19 y vacuna” de mayo a octubre de 2020, que arrojó 90 ensayos de vacunas y 495 ensayos de tratamientos - Brote(s): pandemia mundial de COVID-19 (2020)	- 90 ensayos de vacunas, 88 (97,8 %) excluyeron a las embarazadas - De los 495 ensayos de tratamiento, 350 (70,7 %) excluyeron a las embarazadas - El 25 % de los ensayos requirió anticoncepción	-Tasas de exclusión de mujeres embarazadas en ensayos de vacunas y tratamientos -Requisitos de anticoncepción en participantes aptas para el embarazo	-Estudio limitado a ensayos disponibles en un único registro	Nivel III, Calidad B
16	Malhamé, et al., 2020	Opinión de expertos	-Documento de apoyo a la causa del imperativo moral de incluir a las mujeres embarazadas en los ensayos clínicos -Brote(s): pandemia mundial de COVID-19 (2020)	-Obligación moral de proteger a las mujeres a través de la investigación, en lugar de protegerlas de la investigación.	-Necesidad de adaptar los ensayos clínicos actuales para incluir a las mujeres embarazadas	-Posibilidad de sesgo del autor/a	Nivel V, Calidad B
17	Marban-Castro, et al., 2021	Cualitativo	-Entrevistas semiestructuradas con 24 mujeres embarazadas y 6 profesionales de la salud proveedores de mujeres embarazadas en España entre junio y octubre de 2020 -Brote(s): pandemia mundial de COVID-19 (2020)	-La disposición a participar en un ensayo clínico entre las mujeres embarazadas fue baja (16,7 %) y no dependió del fármaco que se estaba probando -Los temores a los efectos adversos sobre el feto fueron la principal preocupación -Es más probable que participen en ensayos si es percibido un beneficio para la seguridad personal	-Comprensión de los participantes y posible participación en los ensayos clínicos de COVID-19	-Los autores observan la posibilidad de un sesgo de deseabilidad -El tamaño de la muestra es pequeño y está limitado a una región geográfica	Nivel III, Calidad B
18	Minchin, et al., 2023	Revisión sistemática de los estudios observacionales	-Ensayos clínicos de fase II y III de vacunas durante los brotes de 2009 a 2019, búsqueda en 18 registros de ensayos clínicos a nivel mundial, que arrojaron 84 resultados -Brote(s): influenza H1N1, síndrome respiratorio por coronavirus de Oriente Medio, Zika y Ébola	-Las mujeres embarazadas fueron excluidas de más del 80,0 % de los ensayos clínicos de vacunas de emergencia -8 protocolos de ensayos clínicos incluyeron explícitamente a mujeres embarazadas -Menos del 3 % de los estudios monitorearon e informaron embarazos incidentales -Aborda las lagunas en las pautas existentes para la inclusión de mujeres embarazadas	- Criterios de elegibilidad - Resultados del embarazo - Vacíos en las orientaciones existentes	-Limitado a estudios en inglés -Los autores señalan limitaciones y lagunas en los datos	Nivel III, Calidad B
19	Mourad, et al., 2020	Opinión de expertos	-Documento de promoción de la inclusión de mujeres embarazadas en la investigación sobre la COVID-19 -Brote(s): pandemia mundial de COVID-19 (2020)	-42 de los 2100 ensayos clínicos durante la COVID-19 se están llevando a cabo en mujeres embarazadas -Menos del 2% de los ensayos actuales de COVID-19 incluyen mujeres embarazadas -Recomiendan flexibilidad en los procesos de aprobación, colaboración y permitir el consentimiento virtual	-Impacto de la COVID-19 en las investigaciones en curso y nuevas investigaciones sobre el embarazo -Desafíos, estrategias clave son necesarias	-Posibilidad de sesgo del autor/a	Nivel V, Calidad B
20	La ética de la investigación sobre vacunas, epidemias y nuevas tecnologías durante el embarazo (Grupo de trabajo *Prevent* , 2021)	Panel de consenso	-Recomendaciones del Grupo de Trabajo de Prevención -Brote(s): amenazas epidémicas emergentes a nivel mundial	- Sistemas de vigilancia más sólidos con datos pertinentes sobre la salud materna - La investigación debe incluir a las mujeres embarazadas, ser desarrolladas antes de los brotes, incluir recomendaciones específicas para su uso durante el embarazo y tener en cuenta los efectos adversos específicos del embarazo - La conveniencia durante el embarazo debe orientar las decisiones de inversión en vacunas - Consentimiento informado para la participación, incluso durante los brotes, considerar las perspectivas de las mujeres embarazadas y los expertos en salud materna en el desarrollo de vacunas	22 recomendaciones clave para la inclusión de los intereses de las mujeres embarazadas en las vacunas contra patógenos emergentes	-Posibilidad de sesgo del autor/a	Nivel IV, Calidad B
21	Smith, et al., 2020	Observacionales	-Búsqueda online en el registro de ensayos clínicos internacionales de la OMS ^§^ (18 registros) para ensayos relacionados con la COVID-19 en abril de 2020, que arrojó 927 resultados -Brote(s): pandemia mundial de COVID-19 (2020)	-El 52% excluyó explícitamente el embarazo, el 46% no abordó el tema en absoluto -16 ensayos fueron específicos sobre el embarazo, solo 3 fueron ensayos controlados aleatorios -Solo 2 ensayos de medicamentos incluyeron mujeres embarazadas -688 (74,2%) de los ensayos sobre COVID-19 se realizaron en Asia, 128 (13,8%) en Europa y 66 (7,2%) en América del Norte.	- Criterios de elegibilidad para la inclusión/exclusión de mujeres embarazadas, categorizados por región	-Los autores señalan que puede haber otros ensayos relevantes que no se hayan incluido en este análisis -Falta de funciones de búsqueda consistentes en las distintas bases de datos	Nivel III, Calidad B
22	Whitehead & Walker, 2020	Opinión de expertos	-Documento de promoción de la inclusión de mujeres embarazadas en la investigación sobre la COVID-19 -Brote(s): pandemia mundial de COVID-19 (2020)	-Los grupos de apoyadores y las organizaciones profesionales deben presionar para la inclusión segura de las mujeres embarazadas en los ensayos clínicos de terapias y vacunas contra la COVID-19	-Argumentos históricos y éticos para la inclusión	-Posibilidad de sesgo del autor/a	Nivel V, Calidad B
23	Organización Mundial de la Salud, 2020	Panel de consenso	-Directrices para el tratamiento de mujeres embarazadas y lactantes en el contexto de la enfermedad por el virus del Ébola, febrero de 2020 -Brote(s): virus del Ébola a nivel mundial	-Recomienda que se incluya a las mujeres embarazadas en los ensayos clínicos de investigación de vacunas, con disposiciones para el control de la seguridad y la documentación de los casos, y que se haga un seguimiento de las mujeres embarazadas y de sus hijos. -Recomienda ofrecer el uso de terapias en investigación para mujeres embarazadas con Ébola en el contexto de una investigación rigurosa y de conformidad con el protocolo MEURI ^‡^	-Recomendaciones clave para el manejo de mujeres embarazadas en el contexto del Ébola	-Los autores señalan una calidad muy baja de la evidencia existente	Nivel IV, Calidad B

*WHO-ERC = Comité de Revisión Ética de la Organización Mundial de la Salud; ^†^DRC = República Democrática del Congo; ^‡^MEURI = Uso de Monitoreo de Emergencia de Investigación de Intervenciones no Registradas; ^§^OMS = Organización Mundial de la Salud

Se incluyeron en esta revisión 23 estudios publicados entre 2017 y 2023. Se utilizó el modelo de práctica basada en evidencia de enfermería de Johns Hopkins para evaluar el nivel y la calidad de la evidencia de cada artículo^(16)^. Los niveles de evidencia variaron de III a V, y todos los estudios fueron considerados de “buena” o de calidad B.

Dos de los estudios incluidos fueron revisiones sistemáticas de estudios observacionales^(8,17)^. Tres estudios utilizaron una metodología cualitativa, un estudio utilizó encuestas^(18)^ y dos emplearon entrevistas semiestructuradas^(19-20)^. Ocho estudios fueron estudios observacionales y cinco de ellos implicaron búsquedas online en bases de datos de registros de ensayos clínicos^(2,17,21-23)^. Tres estudios fueron paneles de consenso de grupos de trabajo de expertos y organizaciones de salud^(3,24-25)^ y los siete artículos restantes fueron clasificados como Opinión de expertos, que contenían recomendaciones y marcos éticos.

Además, los estudios se clasificaron por los patógenos de la enfermedad: doce estudios analizaron el SARS-CoV-2 (COVID-19), cuatro estudios analizaron la enfermedad por el virus del Ébola (EVE), cuatro estudios analizaron el virus Zika (ZIKA), dos estudios analizaron múltiples patógenos o amenazas epidémicas emergentes en general; un estudio analizó la hepatitis E. Siete estudios revisaron datos de los Estados Unidos, tres de África Occidental, dos de Australia, uno del Reino Unido, uno de la República Democrática del Congo (RDC), uno de España y los ocho estudios restantes analizaron datos de fuentes múltiples o globales.

Al revisar estos estudios, surgieron seis temas principales:

### Exclusión de la investigación durante brotes

Hubo consenso en la literatura revisada en cuanto a que las mujeres embarazadas han sido, en gran medida, excluidas de la investigación durante brotes y pandemias a nivel mundial. En distintas regiones y a lo largo de múltiples brotes de enfermedades, varios artículos encontraron que el embarazo se incluyó específicamente como un criterio de exclusión para los estudios de investigación^(2,4,17,21-23,26-28)^, o se requirió una prueba de embarazo negativa para participar^(2,17,22)^. Las razones de exclusión citadas con más frecuencia incluyeron preocupaciones por la seguridad fetal, evidencia insuficiente sobre la seguridad de la intervención en el embarazo, supuesta falta de voluntad de las mujeres embarazadas para participar o la categorización de las mujeres embarazadas como poblaciones vulnerables que pueden estar más expuestas a los efectos adversos de las intervenciones si comparadas con las adultas no embarazadas^(2,7,18,21)^.

En los estudios que buscaron bases de datos de registros de ensayos clínicos, las tasas de exclusión de mujeres embarazadas estuvieron entre el 52% y el 97,8%^(2,18,21-23)^. Las mujeres embarazadas tenían más probabilidades de que se les negara la elegibilidad para la investigación de desarrollo de vacunas^(17,22,26)^ y en ensayos que prueban tratamientos farmacéuticos^(4,21,23,26)^. Durante el brote de EVE en África Occidental entre 2014 y 2016, las mujeres embarazadas fueron excluidas de casi todos los ensayos terapéuticos y de las vacunas^(2,6-7,17,25-26)^. La descalificación categórica continúa durante la pandemia de COVID-19^(2,4,21-22,27)^. Las mujeres embarazadas fueron excluidas de las investigaciones sobre medicamentos contra la COVID-19 con perfiles de seguridad conocidos durante el embarazo, intervenciones ya utilizadas regularmente durante el embarazo o intervenciones no farmacológicas de bajo riesgo^(4-5,21)^. Pocos ensayos clínicos realizados durante la COVID-19 fueron diseñados específicamente para mujeres embarazadas, y muy pocos de ellos fueron ensayos controlados aleatorios^(2,23)^.

### Preocupaciones éticas y morales

También hubo consenso en la literatura revisada en cuanto a que la descalificación automática de mujeres embarazadas para participar en investigaciones durante brotes y pandemias constituye un problema ético y moral. Varios estudios citaron violaciones de principios éticos, incluida la justicia, el acceso equitativo, el consentimiento informado y la autonomía^(2,6,24,28-29)^. Negar a las mujeres embarazadas la elegibilidad bajo la etiqueta de población vulnerable que requiere protección contra la explotación, niega el beneficio potencial que se ofrece a otros participantes del ensayo y no respeta su capacidad de evaluar de manera autónoma los riesgos y beneficios de la participación y así poder aceptar un consentimiento informado^(2,6)^.

### Necesidad de aceptar la inclusión presunta

En la literatura se ha recomendado de forma consistente un cambio en la inclusión presuntiva para la inclusión predeterminada de mujeres embarazadas en las investigaciones durante brotes y pandemias^(2-6)^. Las mujeres embarazadas solo deben ser descalificadas de participar en investigaciones clínicas si existe una justificación clara para la exclusión, con datos que demuestren que es probable que el riesgo del tratamiento exceda el beneficio para la madre, el feto o ambos^(2,4,6)^. La inscripción voluntaria en una investigación debe basarse únicamente en el consentimiento informado del participante y no requerir el consentimiento adicional de otras personas^(3-4)^. La literatura enfatizó la importancia de la inclusión presuntiva de mujeres embarazadas en investigaciones que prueban terapias con perfiles de seguridad conocidos en el embarazo e intervenciones no farmacológicas de bajo riesgo^(2,21)^.

### Disposición variada a participar en la investigación

La disposición de las mujeres embarazadas a participar en investigaciones durante brotes de enfermedades y pandemias varió en la literatura revisada. La proporción dispuesta a inscribirse en ensayos clínicos varió del 16,7% en un ensayo de tratamiento de COVID-19^(20)^ para 77% para un ensayo hipotético de una vacuna contra el virus del Zika^(18-19)^. El deseo de proteger al bebé de cualquier daño fue citado como el motivador más fuerte tanto para la participación como para la no participación en el ensayo^(18-19)^. Las mujeres tenían más probabilidades de participar en ensayos clínicos si existía evidencia de seguridad proveniente de investigaciones previas, en particular investigaciones sobre mujeres embarazadas, lo que resalta la importancia de recopilar datos y comunicar con precisión los resultados específicos del embarazo^(18-20)^. Las decisiones de participación en los ensayos estuvieron más influenciadas por las recomendaciones de los proveedores de atención a la salud y las autoridades de salud pública, que por las indicaciones de familiares, amigos o del gobierno^(18-19)^. La literatura revisada respaldó que las perspectivas de las mujeres embarazadas eran consideraciones esenciales para diseñar e implementar investigaciones clínicas que involucran participantes embarazadas^(3,7,19-21)^.

### Importancia de la recopilación de datos específicos del embarazo

Otro tema que surgió en la literatura fue la importancia de la recopilación de datos específicos del embarazo, en el contexto de la investigación durante brotes de enfermedades y pandemias. Muchos autores señalaron que había lagunas de datos y oportunidades perdidas para obtener datos de seguridad y eficacia de mujeres embarazadas durante los brotes^(2-4,8,17,24,26,30)^. Los datos de personas no embarazadas, debido a los cambios fisiológicos del embarazo, no pueden generalizarse automáticamente para las mujeres embarazadas; pero, la información sobre el estado y los resultados del embarazo no se recopilan de manera rutinaria o a menudo es insuficiente para el seguimiento a largo plazo^(2,4,17,24,26,30)^. La falta de datos estandarizados sobre vigilancia y sus informes crean barreras para actividades eficaces de prevención y respuesta a los brotes^(3,8,24,30)^. La literatura recomienda el fortalecimiento de los sistemas coordinados de vigilancia de datos y la recopilación sistemática de datos de salud materna para apoyar la inclusión de mujeres embarazadas en futuras investigaciones y orientar las respuestas de la salud pública durante los brotes^(3-4,8,30)^.

### Necesidad de efectuar cambios institucionales y estratégicos globales

Hubo un acuerdo común en toda la literatura para transformar globalmente las estrategias de investigación implementadas para proteger la salud de las mujeres embarazadas durante los brotes^(3,5-7,25,27-28,31-32)^. Se reconoció ampliamente que el cambio de introducir la inclusión presunta requiere cambios de actitudes, de nuevas colaboraciones multidisciplinarias y una reconsideración de los marcos de investigación legales y regulatorios^(3,5,7)^. Se instó a los investigadores clínicos, a los organismos de toma de decisiones y a los financiadores de la investigación a priorizar el desarrollo de terapias y vacunas que sean prometedoras para su uso durante el embarazo, incluyendo opciones alternativas para aquellas personas contraindicadas durante el embarazo, así como la recopilación de datos preclínicos e indicadores específicos del embarazo^(3-4,7)^. Se incentivó a los investigadores clínicos a implementar modificaciones efectivas en las prácticas de investigación durante los brotes para garantizar la continuación segura de la investigación sobre la salud materna^(27,31-32)^. Por último, se encargó a grupos de promoción, a organismos asesores y organizaciones profesionales, que presionaran para incluir a las mujeres embarazadas en ensayos de terapias y vacunas para patógenos emergentes^(3,25,28)^.

## Discusión

Realizar investigaciones sobre mujeres embarazadas es complicado desde el punto de vista ético y logístico e involucra a múltiples partes interesadas, entre ellas los encargados de formular políticas nacionales, las instituciones de investigación, las autoridades reguladoras, los financiadores, los patrocinadores de la investigación, los fabricantes de vacunas y productos farmacéuticos y los organismos de supervisión. Las actuales regulaciones internacionales de investigación son complejas y las preocupaciones sobre la responsabilidad legal influyen en las decisiones de elegibilidad. Normativas vigentes del *Council of International Organizations of Medical Sciences* (CIOMS), de la *Pan American Health Organization* (PAHO), y la Sub-parte B de la *United States Code of Federal Regulations* (45 CFR Parte 46, también conocida como la “*Common Rule*”) apoyan claramente que las mujeres embarazadas deben ser consideradas adultas competentes capaces de dar su consentimiento informado para participar en ensayos que les ofrezcan un beneficio potencial^(3,33-34)^. Adicionalmente, CIOMS y la Política Federal de Protección de Sujetos Humanos actualizó sus políticas en 2016 y 2019, respectivamente, para reconocer que el embarazo por sí solo no califica a alguien como parte de una “población vulnerable” en el contexto de participar de una investigación^(3)^.

La literatura citó algunos ejemplos importantes de inclusión exitosa de mujeres embarazadas en ensayos clínicos durante brotes que pueden estudiarse, para orientar futuras directrices y prácticas de investigación. En 2019, el Grupo de Expertos en Asesoramiento Estratégico (GEAE) de la OMS, recomendó que los ensayos clínicos de las vacunas contra el virus del Ébola en la República Democrática del Congo incluyan a mujeres embarazadas y lactantes, y que las participantes embarazadas sean vigiladas de cerca para detectar efectos adversos hasta el nacimiento de sus bebés^(7)^. Cuatro meses después, el Comité Nacional de Ética de la Escuela de Salud Pública de la Universidad de Kinshasa aprobó la administración de esta vacuna a las mujeres embarazadas después del primer trimestre^(7)^. Sin embargo, pasaron más de diez meses desde el inicio del brote de Ébola de 2020, en la República Democrática del Congo, hasta que la vacuna pudiese ser administrada a las mujeres embarazadas^(7)^.

Otro ejemplo es el ensayo de evaluación aleatoria de la terapia contra la COVID-19 (RECOVERY) en el Reino Unido en 2020, que probó tratamientos para pacientes ingresados en el hospital con neumonía por COVID-19 e incluyó a mujeres embarazadas y lactantes^(35)^. El diseño del estudio utilizó datos existentes de lopinavir-ritonavir para el tratamiento del HIV durante el embarazo e hidroxicloroquina para el tratamiento del lupus durante el embarazo^(5)^. Los investigadores también adaptaron algunas intervenciones para uso aceptable en participantes embarazadas, como sustituir la dexametasona por prednisolona o hidrocortisona para evitar efectos fetales adversos^(5)^. Se superaron las barreras regulatorias al proporcionar revisiones de seguridad realizadas por expertos en salud materna. Un autor calificó este estudio como “un ejemplo de éxito en materia de equidad para las mujeres embarazadas”^(5)^.

a) Con base en la evaluación crítica y la síntesis de la evidencia en la literatura, se han formulado las siguientes recomendaciones resumidas para realizar investigaciones clínicas en mujeres embarazadas, durante brotes y pandemias a nivel mundial. Cabe señalar que estas recomendaciones se aplican a todas las personas embarazadas independientemente de su identidad de género.
b) Las mujeres embarazadas solo deben ser excluidas de participar en investigaciones clínicas durante brotes con una justificación clara a partir de datos que demuestren que los riesgos propuestos de una intervención probablemente excedan los beneficios esperados.
c) Los comités de revisión ética y las organizaciones profesionales pertinentes deben solicitar modificaciones al protocolo y abogar por la revocación de decisiones que excluyen mujeres embarazadas de los ensayos clínicos durante brotes, sin una justificación clara.
d) Los posibles riesgos y beneficios potenciales de la participación en ensayos clínicos, durante brotes, deben explicarse claramente a las participantes embarazadas. Las decisiones de participación deben basarse en el principio del consentimiento informado y no deben requerir el consentimiento de ningún otro actor.
e) Se deben desarrollar, fortalecer y utilizar sistemas estandarizados de recopilación de datos que incluyan indicadores específicos del embarazo para la recopilación oportuna de datos durante los brotes de enfermedades.
f) La participación de la comunidad, la educación del paciente y la comunicación de la evidencia existente a las posibles participantes embarazadas, deben tener prioridad por parte de proveedores de salud confiables, antes y durante los ensayos clínicos en el contexto de un brote de enfermedad.
g) El desarrollo de intervenciones aceptables para uso en el embarazo durante los brotes y los incentivos financieros deben alinearse con esta prioridad.
h) Se deben priorizar los datos de los ensayos preclínicos necesarios para la futura inclusión de mujeres embarazadas en ensayos clínicos durante un brote.
i) Los investigadores deben consultar con expertos en salud materna sobre la inclusión o exclusión de mujeres embarazadas en ensayos clínicos durante los brotes.
j) Las perspectivas de las mujeres embarazadas deben tenerse en cuenta al diseñar e implementar ensayos clínicos en los que puedan inscribirse mujeres embarazadas.

Los investigadores deben implementar modificaciones basadas en evidencia, en los procesos de investigación, para continuar realizando investigaciones perinatales de manera segura durante los brotes de enfermedades.

Este artículo contribuye al avance del conocimiento científico al destacar la exclusión sistemática de las mujeres embarazadas de las investigaciones durante los brotes de enfermedades e identificar las barreras éticas, logísticas y metodológicas para su inclusión. Además, sintetiza los hallazgos clave de la literatura para abogar por un cambio de paradigma indicando la inclusión presunta de mujeres embarazadas en la investigación clínica. Por último, propone recomendaciones basadas en evidencias para fundamentar estrategias futuras destinadas a mejorar los resultados de salud materna y fetal durante emergencias de salud pública.

Esta revisión de la literatura tiene como objetivo sintetizar la literatura disponible sobre la realización de investigaciones en mujeres embarazadas durante brotes y pandemias a nivel mundial. Si bien los hallazgos proporcionaron información valiosa sobre el tema, los estudios revisados tienen limitaciones importantes. La mayor parte de la evidencia provino de países de altos ingresos, lo que limita la generalización en países de ingresos medios y bajos. Los niveles de evidencia fueron relativamente bajos y no se identificaron estudios experimentales o cuasi experimentales. Estos hallazgos resaltan la necesidad de realizar investigaciones de alta calidad que utilicen diseños experimentales que aborden este tema. Además, la mayoría de los artículos se centraron en los hallazgos de un solo brote de enfermedad, en una región geográfica particular, que podrían no ser generalizados a todos los brotes de enfermedades y pandemias a nivel mundial.

## Conclusión

El alcance de esta revisión destaca la exclusión sistémica de las mujeres embarazadas de la investigación clínica durante los brotes de enfermedades, lo que revela importantes preocupaciones éticas, lagunas de conocimiento y oportunidades perdidas para promover la salud materna y fetal. Entre las principales lagunas de conocimiento identificadas se incluyen la falta de recopilación estandarizada de datos específicos del embarazo, la investigación limitada que abordó la seguridad y eficacia de las intervenciones durante el embarazo y la falta de estudios en países de ingresos bajos y medios. A través de la síntesis de 23 estudios sobre múltiples brotes, esta revisión identificó barreras como criterios de inclusión inconsistentes y datos preclínicos inadecuados, los que dificultan la toma de decisiones clínicas basada en evidencia. Al sintetizar la evidencia existente se pudo formular recomendaciones viables e identificar lagunas de conocimiento; esta revisión proporciona una base para futuros esfuerzos encaminados a avanzar en la investigación sobre salud materna durante emergencias de salud pública.
